# Meta-Substituted Asymmetric Azobenzenes: Insights into Structure–Property Relationship

**DOI:** 10.3390/molecules29091929

**Published:** 2024-04-23

**Authors:** Anna Laura Sanna, Tatiana Pachova, Alessandra Catellani, Arrigo Calzolari, Giuseppe Sforazzini

**Affiliations:** 1 Department of Chemical and Geological Sciences, Università degli Studi di Cagliari, SS 554, Bivio per Sestu, 09042 Cagliari, Italy; 2 Laboratory of Macromolecular and Organic Materials, Institute of Material Science and Engineering, Ecole Polytechnique Federale de Lausanne (EPFL), 1015 Lausanne, Switzerland; 3 CNR-NANO, Istituto Nanoscienze, Via Giuseppe Campi, 213, 41125 Modena, Italy

**Keywords:** azobenzene, *meta*-substitution, molecular switch, structure–property relationship, asymmetric functionalization

## Abstract

This article presents a comprehensive investigation into the functionalization of methoxyphenylazobenzene using electron-directing groups located at the *meta* position relative to the azo group. Spectroscopic analysis of *meta*-functionalized azobenzenes reveals that the incorporation of electron-withdrawing units significantly influences the absorption spectra of both *E* and *Z* isomers, while electron-donating functionalities lead to more subtle changes. The thermal relaxation process from *Z* to *E* result in almost twice as prolonged for electron-withdrawing functionalized azobenzenes compared to their electron-rich counterparts. Computational analysis contributes a theoretical understanding of the electronic structure and properties of *meta*-substituted azobenzenes. This combined approach, integrating experimental and computational techniques, yields significant insights into the structure–property relationship of *meta*-substituted asymmetrical phenolazobenzenes.

## 1. Introduction

Azobenzene is a widely studied molecule that has garnered significant attention in diverse fields including chemistry, physics, materials science, biology, and pharmacology [1,2,3]. Its unique photoresponsive properties, arising from its ability to undergo *E*/*Z* isomerization upon absorption of light, have made it a popular choice for designing light-responsive molecular switches [4,5,6]. The facile and reversible nature of the photoisomerization process, which can be triggered by different wavelengths of light, has enabled a plethora of applications for azobenzene-containing systems, including photo-controlled cargo delivery [7,8,9,10], surface patterning [11,12,13,14], mechanical actuators [15,16,17,18,19], and biological process regulators [20,21,22]. The *E*/*Z* photoisomerization of azobenzenes occurs by electron transition from an occupied π molecular orbital (MO) to an unoccupied π* MO localized on the N=N bond. At the same time, the empty π* MO of the double bond can be accessed from the non-bonding orbital (n) of the N lone electron pairs. These π–π* and n–π* transitions give rise to distinct and well-separated absorption bands in the spectra of azobenzenes. Due to the higher energy levels of n electrons compared to π electrons, the excitation energy required for n–π* transitions is generally lower. Consequently, the absorption band corresponding to the n–π* excitation is commonly red-shifted relative to the π–π* band. Typically, the π–π* transition is observed as an intense band around 320 nm, while the n–π* transition is observed as a weak broad band centered around 450 nm. The conversion of azobenzenes from the stable isomer *E* to the metastable isomer *Z* is exclusively initiated by light, while the reverse interconversion can take places either through photochemical or thermal processes. The spectroscopic characteristics and the isomerization behavior of azobenzenes are primarily governed by the azo group, along with the nature and the position of substituents on the two phenyl rings. Within this context, considerable effort has been put into reducing the spectral overlap between π–π* and n–π* transition bands, and in tailoring the *Z→E* thermal relaxation time for numerous uses. Over the years, extensive and comprehensive studies have been undertaken to achieve these objectives by functionalizing azobenzenes at both their *ortho* and *para* positions relative to the N=N group [1,4,5,6]. Substituting azobenzene’s *para* position (4-position) with electron-donating groups (e.g., hydroxy, amino, etc.) induces a red-shift in the π–π* band (400–450 nm), while the n–π* band remains unaffected, causing convergence and partial overlap of the two bands. 

**Figure 1:**
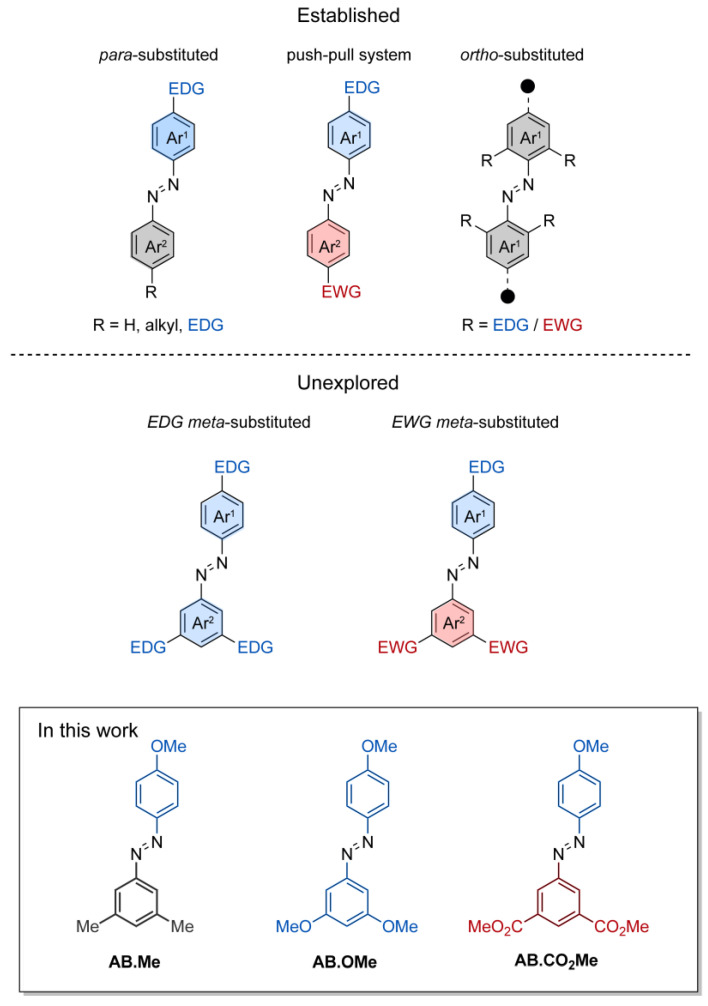
Schematic representation of functionalized azobenzenes. Functionalizations in either one or two *para* positions, functionalization in push–pull system, and functionalization in *ortho* positions (top half); functionalization in *meta* (bottom half); and the compound investigated in this work (framed).

This functionalization is frequently employed to introduce a reactive site into azobenzenes for attaching solubilizing chains and linker, and it has been successfully used to develop photoswitchable surfactants [23,24] and a photo-responsive assembly monolayer on surfaces and on nanoparticles [25,26]. Introduction of strong electron-withdrawing groups (e.g., cyano, nitro) at the second azobenzene *para* position (4′-postion) forms pseudo-stilbene-type azobenzenes (Figure 1). These molecules exhibit overlapping π–π* and n–π* absorption bands within the visible spectrum, reducing *E*/*Z* isomerization yield due to concurrent reverse *Z*/*E* isomerization triggered by the same wavelength. Pseudo-stilbene azobenzenes find commonly applications in nonlinear optics [27] and light-responsive polymeric materials [28]. Functionalization at the *para* position also plays a role in reducing the half-life of *Z* isomers. This reduction is notable, typically decreasing on the order of seconds for 4-substituted azobenzenes and milliseconds for 4,4′-azobenzenes, in contrast to the hours required for the original azobenzene configuration. 

The concept of *ortho* substitution, introduced by Hecht and Woolley (Figure 1), strategically places electron-directing groups at all four *ortho* positions of azobenzene to form tetra-*ortho*-substituted azobenzenes. Substituting electron-withdrawing (e.g., fluorine, chlorine, bromine) [29] or electron-donating groups (e.g., methoxy, S-alkyl) [30] separates n–π* bands of isomers, enhancing *Z* isomer stability from hours to years. Given their distinct band separation, red-shifted spectrum, and the slow thermal relaxation of the *Z* isomers, tetra-*ortho* azobenzenes have emerged as promising candidates for effectively controlling the structure and functionality of biomolecules through photoactivation [31,32].

Substitutions at the *meta* positions of azobenzenes are a common occurrence. *Meta*-substitutions are frequently employed to serve structural purposes, enabling the creation of intricate molecular architectures. Substituents at the *meta* positions of azobenzene have been utilized in the development of light-responsive elastomers [33], molecular cages [34], and organic semiconductors [16]. However, there exists a notable gap in the understanding regarding the profound influence that electron-directing groups impart on the photoswitching and thermal relaxation dynamics of *meta*-substituted azobenzenes. While these functionalities are anticipated to not directly influence the electronic delocalization along the whole molecular π system by mesomeric effects, their spatial arrangement and electron localization on each individual phenyl ring may play a crucial role in the physical properties of azobenzenes. To date, the majority of studies focusing on understanding the effects of functionalization in the *meta* position have been primarily dedicated to symmetric azobenzenes containing alkyl functionalities or halogens [35,36]. With the aim of expanding existing knowledge of the structure–activity relationship of azobenzene, we undertook a comprehensive investigation involving three distinct types of substituents located at the *meta* positions of alkoxyl-azobenzenes, Figure 1. These compounds where investigated spectroscopically to provide information on the photoswitching activity and the thermal relaxation, as well as by computational approach to pinpoint the electronic characteristics of their *E* and *Z* isomers.

## 2. Results and Discussion

### 2.1. Design and Synthesis

The design of the azobenzene was envisioned to incorporate two methoxy groups as electron-donating functionalities, two carbonyl groups as electron-withdrawing functionalities, and two methyl groups as a non-electron-directing reference compound. The inclusion of the hydroxyl functionality on a phenyl ring was intended to complement the investigation of 4-substituted electron-directing azobenzenes. The compounds under investigation were synthesized by reaction of phenol with the azonium salts of *meta* di-substituted amino benzenes, resulting in the production of the corresponding *meta*-di-substituted hydroxyl azobenzenes. Subsequently, these were subjected to alkylation through iodomethane, leading to the desired compounds **AB.Me**, **AB.OMe**, and **AB.CO_2_Me** (Figure 1; for more details, refer to Section 3). The alkylation of the hydroxyl group was conducted in anticipation of potential applications, requiring the insertion of solubilizing groups, linker to connect polymers, and biomolecules. 

### 2.2. UV–vis Characterization

**Figure 2:**
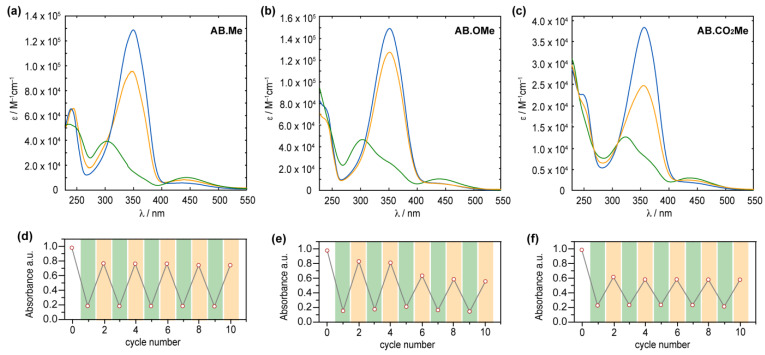
Absorption spectra of **AB.Me** (**a**), **AB.OMe** (**b**), and **AB.CO_2_Me** (**c**) in tetrahydrofuran (THF) solution; *E* isomer (blue), *Z* isomer (green), and photostationary state PSS450 nm (orange). Measured absorbance at λ_max_ for **AB.Me** (**d**), **AB.OMe** (**e**), and **AB.CO_2_Me** (**f**) in THF solutions by alternately irradiating the sample at λ = 365 nm (green) and λ = 450 nm (orange) in repeated switching cycles.

The obtained compounds were initially investigated by UV–vis absorption spectroscopy. The absorption spectrum of *meta*-methylated azobenzene in its E-form displays a characteristic pattern with two separate bands. The first, a prominent peak around 350 nm, derives from π–π* excitation, while the second, a weaker band centered at 444 nm, corresponds to the n–π* transition (Figure 2a). Additionally, a further band at higher energy is observed around 237 nm, which is associated with the π–π* transition resulting from molecular orbitals primarily localized on one of the two aromatic rings [15,35]. The value measured for the first two bands are in good agreement with those reported in literature for alkoxylazobenzens [23,25,26], so highlighting that the alkylation on *meta* position has a negligible effect on the absorption spectra features. Replacement of the two methyl units with two electron donating methoxy groups induced modest changes to all the three bands. 

Compound **AB.OMe** exhibits a λ_max_ at 353 nm deriving from the π–π* transition, and a signal at 440 nm, for the n–π* counterpart. In this case, however, the signal correlated with the high-energy π–π* is blue-shifted to overlap the solvent cut off area, resulting in undefined band. The limited spectral variation deriving from the introduction of electron donating functionalities at the *meta* position of azobenzenes suggests that the electronic delocalization over the molecules remains largely unaffected (see also molecular orbital analysis below). Differently, introduction of two carbonyl electron-withdrawing groups at the *meta* positions influence more effectively the spectra signature of the azobenzene. The resulting functionalized **AB.CO_2_Me** exhibit a bathochromic shift of approximately 8 nm in the low-energy π–π* transition band, with a λ_max_ at 358 nm, and a red-shift of approximately 14 nm in the high-energy π–π* transition, centered at 251 nm. Interestingly, the n–π* transition signal undergoes a hypsochromic shift of around 7 nm compared to **AB.Me**. In a less pronounced manner, this scenario is reminiscent of what is observed for azobenzene within a push–pull system, where the primary band arising from π–π* transition shifts towards longer wavelengths, while the n–π* band experiences minimal changes. The spectral shift observed for the high excitation band is of particular interest as this can be exploited to promote the *Z*→*E* isomerization when the n–π* transition band is not easily accessible. This situation has been observed in various scenarios, including constrained azobenzenes macrocycle [15], intricate structures with overlapping absorption spectra [16], and E-4-aminobenzenes [36]. The redshift of the high-energy π–π* band can offer a distinct advantage by reducing its overlap with the absorption spectra of the solvents used in the sample preparation process, thus facilitating easier access to this band. Upon irradiation at 365 nm, the E isomers of azobenzenes are converted to their corresponding *Z* counterparts (Figure 2). The resulting absorption spectra exhibit the characteristic signature of azobenzene in its *Z* conformation, featuring an approximate 87% reduction in intensity for the π–π* band in the case of **AB.Me**, an 83% reduction for **AB.OMe**, and an 79% reduction for **AB.CO_2_Me**, accompanied by an enhancement in the n–π* band. Significantly, the π–π* band in the Z isomer of **AB.CO_2_Me** exhibits a distinct bathochromic shift of approximately 21 nm compared to **AB.Me** and **AB.OMe**. 

It is noteworthy that the introduction of electron-withdrawing groups in the *meta* position predominantly affects the electronic transitions associated with the *Z* isomer, while exerting a negligible impact on those of the *E* form. All three compounds can undergo back-isomerization to the *E* isomer through light-assisted processes. When irradiated at 450 nm, *meta*-substituted azobenzenes, **AB.Me**, **AB.OMe**, and **AB.CO_2_Me**, exhibit a recovery of approximately ~75%, ~85%, and ~60%, respectively, in the initial absorption intensity of the *E* isomer. This process reaches a photostationary state (PSS), as illustrated in Figure 2. The photoisomerization between the two isomers during alternating irradiation cycles at λ = 365 nm and λ = 450 nm, respectively, did not lead to any noticeable degradation for the compounds **AB.Me** and **AB.CO_2_Me**. This highlights that the introduction of electron withdrawing groups at the *meta* position does not compromise the photochemical stability of the azobenzene structure. However, when subjecting **AB.OMe** to alternating cycles of irradiation, a gradual decrease in both the *E* and *Z* absorption intensities is observed. This phenomenon suggests the presence of a competitive process alongside light-induced isomerization, possibly indicating a gradual photodegradation.

### 2.3. Thermal Relaxation

**Figure 3:**
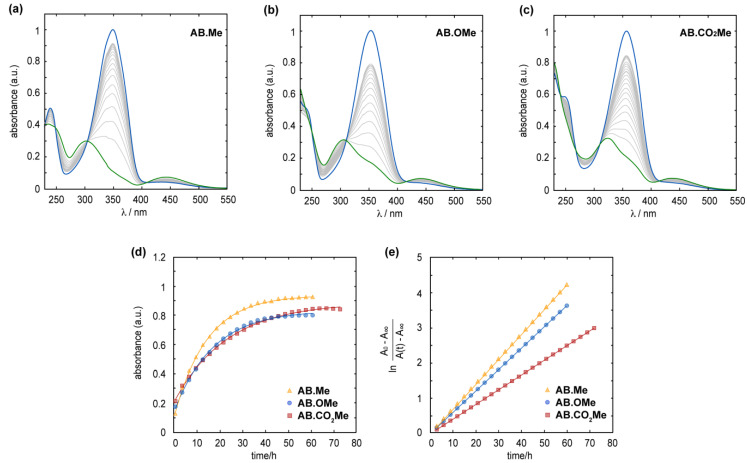
Measured absorption spectra for the *Z* (blue line) to *E* (green line) thermal relaxation of **AB.Me** (**a**), **AB.OMe** (**b**), and **AB.CO_2_Me** (**c**) in diluted THF solutions at 25 °C. Measured absorbance at λ_max_ for **AB.Me** (yellow triangle), **AB.OMe** (blue circle), and **AB.CO_2_Me** (red square) in THF solutions over time; a logarithmic fit (**d**) and a linear fit were used to estimate relaxation rate constants (**e**).

In all three compounds, the metastable Z isomer can spontaneously revert to the *E* form through thermal relaxation. The *Z*-to-*E* isomerization process was observed to proceed at a slower rate in electron-withdrawing functionalized azobenzenes compared to the alkylated and alkoxylated counterpart. At a temperature of 25 °C, the half-life (τ_1/2_) for the *Z*-to-*E* isomerization was approximately 8.5 h for **AB.Me** and **AB.OMe,** in contrast to approximately 9.5 h for **AB.CO_2_Me**. Complete back isomerization of m-substituted azobenzenes is achieved within approximately 1.8 days for **AB.Me** and **AB.OMe**, whereas an additional day is required for **AB.CO_2_Me**, totaling 2.7 days (see Figure 3). The analysis of the kinetics of the back isomerization from *Z* to *E* underscores the varying rates at which each compound undergoes thermal relaxation, with a trend following the order of **AB.Me** > **AB.OMe** > **AB.CO_2_Me**, with a constant rate (k_R_) of 7.0 × 10^−2^ h^−1^, 5.9 × 10^−2^ h^−1^, and 4.3 × 10^−2^ h^−1^, respectively. 

A plausible explanation for this phenomenon can be attributed to the enhanced stability of the *Z* isomer conferred by the electron-directing groups. This increased stability is primarily ascribed to the electronic effects of the electron-directing functionality, as opposed to the bulkiness of the group, which would otherwise promote the destabilization of the *Z* isomers. This scenario is especially pronounced with the carbonyl group, being the largest functionality among the three, yet exhibiting a slower thermal relaxation rate. The recent literature on azobenzenes suggests that the thermal isomerization mechanism involves both singlet and triplet electronic states, engaging in inversion and rotation processes [37,38]. Notably, the rotation mechanism through triplet states has been recognized as the predominant.

The rotational process encompasses out-of-plane CNNC-torsional motion, facilitated by the -N=N- π-bond rupture, ultimately reaching the CNNC-torsional angle characteristic of a nearly perpendicularly twisted conformation [37]. In such a scenario, the isomerization of azo aromatic groups through rotation at its transition state can be conceptualized as a phenylnitrenium ion. Investigation on the impact of substitution with electron-directing groups in the *meta* position of phenylnitronium ions has demonstrated a substantial influence on the stability of the triplet state [39]. The process is attributed to the transfer of an electron from a nonbonding orbital on the electron-directing substituent(s) to the out-of-plane nonbonding orbital on the nitrenium ion center. In essence, this mechanism could theoretically be applied to any species featuring a strong electron acceptor and a strong donor conjugated with non-disjoint π orbitals [39]. As a result, we hypothesize that while *meta*-functionalization modestly affects the π conjugation delocalization of azobenzenes, it nonetheless contributes to the stabilization of the triplet states that govern the rotation mechanism during the thermal isomerization.

### 2.4. Computational Studies

**Figure 4:**
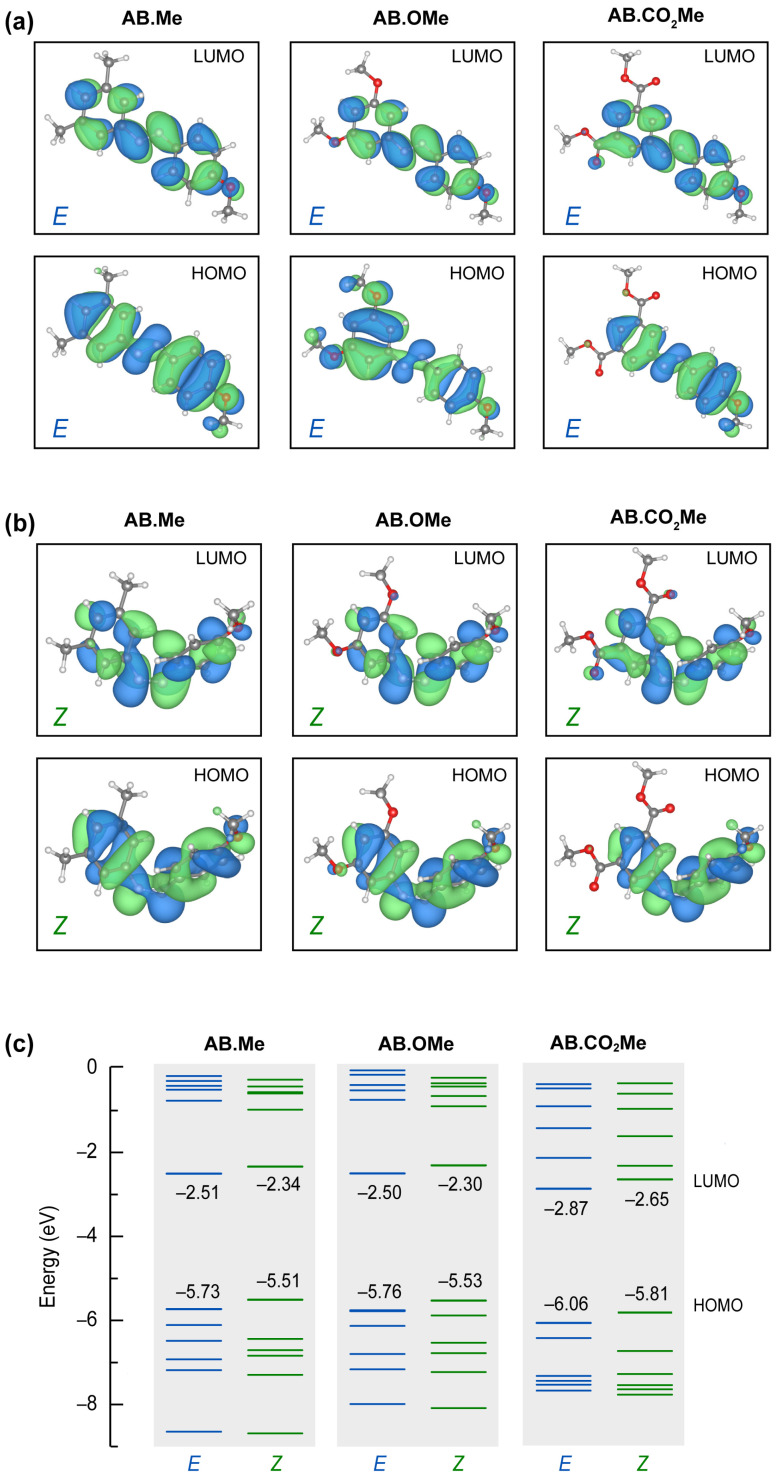
Calculated molecular orbitals HOMO and LUMO for all the *E* (**a**) and *Z* (**b**) isomers studied in this work, and corresponding energy levels (**c**).

To gain insight into the stability of the *E* and *Z* isomers, we analyzed their frontier HOMO and LUMO orbitals resulting from first-principles calculations based on density functional theory (DFT). The results for the *E* and *Z* conformations are summarized in Figure 4 (see Section S5 of Supplementary Materials for further details). 

All E isomers have a p-character spread over the phenyl groups, with minor or no contributions on the attached groups, regardless their electron attractive/withdrawing character. The structural folding in *Z* configurations put the aromatic rings closer, promoting an enhanced p-overlap between them. The LUMO states are hardly affected by the different functionalization for all the geometries. On the other hand, HOMO states are more sensitive to the position of the attached group and isomerization. In particular, the HOMO state for the *E* configuration of **AB.OMe** differs from the other orbitals shown in Figure 4a for both symmetry and for the contribution of the attached methoxy groups. The methyl and the carbonyl terminations do not contribute to the HOMO states of the corresponding molecules. These characteristics of the **AB.OMe** HOMO states change in the *Z* configurations, where both the symmetry and the methoxy contribution results more similar to other azobenzenes. The overall similarities of the frontier orbitals concur with the orbital transition analysis deduced from the absorption spectra. The different electron donor/acceptor character of the attached groups changes the overall polarization of the molecules. The specific dipolar interaction affects the energy position of the molecular orbitals and, therefore, the different energy transitions (i.e., peak energy) detected in the optical spectra. As shown in Figure 4c, **AB.Me** and **AB.OMe** have very similar orbital energy distributions. The electron withdrawing character of **AB.CO_2_Me** accumulates charge in one part of the molecule increasing the internal dipole. The effect is a downshift of frontier orbitals (for both *E* and *Z* configuration) of ~30 meV (see Section S5 of Supplementary Materials for details). We further remark an overall redistributions of the entire energy spectrum (especially HOMO-3, HOMO-4, LUMO+1, and LUMO+2 states) of **AB.CO_2_Me** and increased difference between the E and Z isomers, not observed for the other two azobenzene moieties. This is agrees with the stability analysis described above. 

### 2.5. ^1^H-NMR Analysis

**Figure 5:**
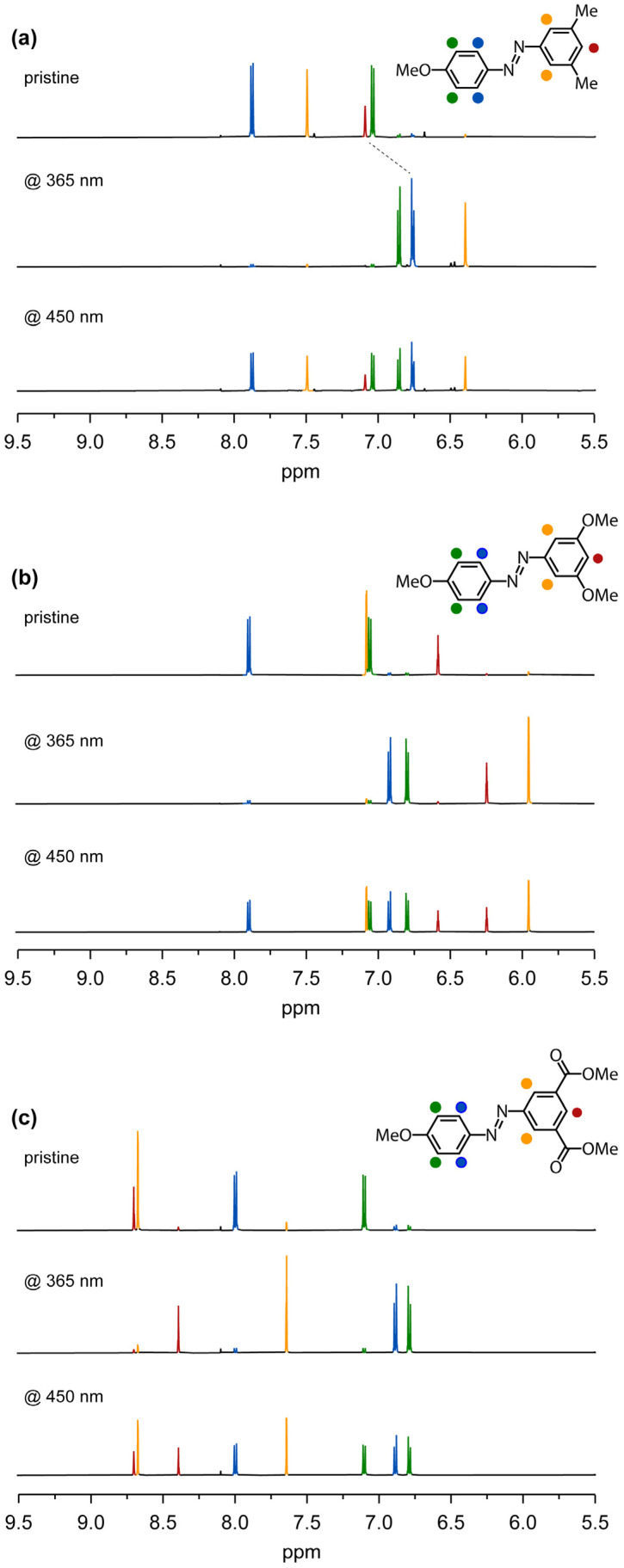
Aromatic region of the ^1^H-NMR spectrum of compounds **AB.Me** (**a**), **AB.OMe** (**b**), and **AB.CO_2_Me** (**c**) in THF-d8. Pristine (top spectrum), after irradiation at 365 nm (middle spectrum), and after irradiation at 450 nm (bottom spectrum). ^1^H-NMR spectra in chloroform-d are reported in the Supplementary Materials.

Finally, to assess the amount of *E* and *Z* isomers generated after photoexcitation at both 365 nm and 450 nm, we utilized nuclear magnetic resonance spectroscopy. Pristine E isomers display distinct spectral signatures. A comparison of the ^1^H-NMR spectral profiles of **AB.OMe** and **AB.CO_2_Me** with that of the reference compound **AB.Me** in Figure 5 reveals a significant displacement of the aromatic proton peaks within the *meta*-substituted benzene ring. 

Specifically, in the reference compound **AB.Me**, the protons of the dimethylphenyl moiety are positioned at 7.50 and 7.09 ppm, respectively. Upon substituting the methyl group with the electron-donating group, the protons experience a shielding effect, resulting in a downfield shift to 7.07 and 6.57 ppm. Conversely, the introduction of carbonyl functionalities in place of the methyl group induces a deshielding effect, shifting the proton peaks to a lower magnetic field at 8.69 and 8.67 ppm. Upon irradiation at 365 nm, all the compounds adopt their respective Z forms. The ^1^H-NMR spectral profiles reveal the positions of protons in **AB.Me** at 6.75 and 6.40 ppm, in **AB.OMe** at 6.23 and 5.94 ppm, and in **AB.CO_2_Me** at 8.38 and 7.64 ppm. Upon comparing the ^1^H-NMR signal intensities of the E and Z isomers, we determined that photoisomerization of **AB.Me** occurs with a yield of 93%, while **AB.OMe** and **AB.CO_2_Me** exhibit yields of 90% and 89%, respectively. Irradiation at 450 nm facilitates the generation of the initial E isomer, ultimately attaining a PSS with yields of 51%, 49%, and 51% for **AB.Me**, **AB.OMe**, and **AB.CO_2_Me**, respectively.

## 3. Materials and Methods

### 3.1. General Experimental Details

All solvents and reagents were used as commercially supplied, without further purification. All the solvents (dichloromethane, ethyl acetate, and petroleum ether 40–60 °C) were purchased from Carlo Erba Reagents. Iodomethane was purchased from Sigma Aldrich, St. Louis, MO, USA; dimethylformamide (DMF, 99.0%) and 3,5-Dimethylaniline were purchased from Fluorchem, Glossop, UK; concentrated hydrochloric acid 37%, phenol, NaNO_2_, NaOH, and K_2_CO_3_ were purchased from Carlo Erba Reagents, Cornaredo, Italy; 3,5-Dimethoxylaniline was purchased from ABCR and dimethyl 5-aminoisophthalate was purchased from TCI. Reactions were monitored by thin-layer chromatography (TLC) performed on alugram precoated silica sheets, 0.2 mm plates (MN), and compounds were visualized under UV light (254 nm or 365 nm), depending on the substrate. Column chromatography was performed on silica gel 60A (particle size 40–63 μm, Carlo Erba) using positive air pressure. ^1^H and ^13^C liquid NMR spectra were recorded on a Bruker Advance III HD 600 MHz NMR spectrometer at 298 K. Deuterated chloroform was used for preparing the NMR sample and purchased from Carlo Erba Reagents. Chemical shifts (δH and δC) are expressed in parts per million (ppm) relative to the residual solvent peak and, for proton NMR, shown as follows: chemical shift, multiplicity (s = singlet; d = doublet; t = triplet; q = quartet; m = multiplet), coupling constant (J, Hz), and integration. Mass analyses were performed using a Exploris 240 LC/GC-Orbitrap FTMS mass spectrometer. 

### 3.2. Synthetic Procedures and Characterization

*Compound **AB-OH.Me**, (E)-4-((3,5-dimethylphenyl)diazenyl)phenol.* This compound was synthesized as reported in the literature [15]. Concentrated hydrochloric acid (2 mL) was added to a suspension of 3,5-dimethylaniline Ph-NH_2_.Me (0.606 g, 5.00 mmol, 1 eq) in water (20 mL). The mixture was cooled to 0 °C in an ice bath and stirred vigorously. A solution of NaNO_2_ (0.380 g, 5.50 mmol, 1.1 eq) in water (3 mL) was then added dropwise to the cooled suspension, and the mixture was stirred for 1 h. In a separate flask, NaOH 5 M (0.999 g, 25.0 mmol, 5 eq) was added to a solution of phenol (0.565 g, 6.00 mmol, 1.2 eq) in water (2 mL), and cooled to 0 °C. The former mixture was then slowly transferred to the solution of phenol and vigorously stirred for 1 h at 0 °C. The resultant precipitate was filtered with vacuo and purified by silica chromatography (dichloromethane/ethyl acetate 9.5:0.5) to give an orange powder (0.404 g, 35.7%). ^1^H NMR (600 MHz, CDCl_3_): δ = 7.86 (d, *J* = 8.8 Hz, 2H), 7.49 (s, 2H), 7.09 (s, 1H), 6.94 (d, *J* = 8.8 Hz, 2H), 5.08 (s, 1H, OH), 2.41 (s, 6H). ^13^C NMR (151 MHz, CDCl_3_): δ = 158.15, 153.06, 147.47, 138.85, 132.32, 125.00, 120.51, 115.90, 21.47. HRMS (ESI, positive mode) *m*/*z* calculated for C_14_H_15_N_2_O [M + H]^+^: 227.1174; found: 227.1179 [40].

*Compound **AB-OH.OMe**, (E)-4-((3,5-dimethoxyphenyl)diazenyl)phenol.* This compound was synthesized adapting a procedure from the literature [40]. Concentrated hydrochloric acid (1.3 mL) was added to a suspension of 3,5-dimethoxyanline Ph-NH_2_.OMe (0.500 g, 3.26 mmol, 1 eq) in tetrahydrofuran (5 mL). The mixture was cooled to 0 °C in an ice bath and stirred vigorously. A solution of NaNO_2_ (0.247 g, 3.59 mmol, 1.1 eq) in 1.95 mL of water was then added dropwise to the cooled suspension, and the mixture was stirred for 1 h. In a separate flask, NaOH 5 M (0.652 g, 16.3 mmol, 5 eq) was added to a solution of phenol (0.368 g, 3.91 mmol, 1.2 eq) in 2.6 mL of water, and cooled to 0 °C. The former mixture was then slowly transferred to the solution of phenol and vigorously stirred for 1 h at 0 °C. The reaction mixture was extracted with dichloromethane, the organic layers collected, dried over Na_2_SO_4_, filtered, and solvent removed with vacuo. The crude product was purified by silica chromatography using a gradient eluent (dichloromethane 100% → ethyl acetate 100%) to obtain the desired product as a red dark powder (0.377 g, 44.8%). ^1^H NMR (600 MHz, CDCl_3_) δ 7.88 (d, *J* = 8.8 Hz, 2H), 7.08 (s, 2H), 6.95 (d, *J* = 8.8 Hz, 2H), 6.57 (s, 1H), 5.17 (s, 1H, OH), 3.87 (s, 6H). ^13^C NMR (151 MHz, CDCl_3_) δ 161.22, 158.49, 154.68, 147.16, 125.22, 115.96, 103.55, 100.81, 55.75. HRMS (ESI, positive mode) *m*/*z* calculated for C_14_H_15_N_2_O_3_ [M + H]^+^: 259.1083; found: 259.1082.

*Compound **AB-OH.CO_2_Me**, (E)-dimethyl 5-((4-hydroxyphenyl)diazenyl)isophthalate.* This compound was synthesized adapting a procedure from the literature [40]. Concentrated hydrochloric acid (0.95 mL) was added to a suspension of dimethyl 5-aminoisophthalate Ph-NH_2_.CO_2_Me (0.500 g, 2.39 mmol, 1 eq) in tetrahydrofuran (5 mL). The mixture was cooled to 0 °C in an ice bath and stirred vigorously. A solution of NaNO_2_ (0.182 g, 2.63 mmol, 1.1 eq) in 1.42 mL of water was then added dropwise to the cooled suspension, and the mixture was stirred for 1 h. In a separate flask, NaOH 5 M (0.476 g, 11.9 mmol, 5 eq) was added to a solution of phenol (0.270 g, 2.87 mmol, 1.2 eq) in 1.92 mL of water, and cooled to 0 °C. The former mixture was then slowly transferred to the solution of phenol and vigorously stirred for 1 h at 0 °C. The reaction mixture was extracted with ethyl acetate, the organic layers collected, dried over Na_2_SO_4_, filtered, and solvent removed with vacuo. The crude product was purified by silica chromatography using dichloromethane/ethyl acetate (9.5:0.5) as an eluent, to obtain the desired product as bright orange powder (0.515 g, 68.6%). ^1^H NMR (600 MHz, CDCl_3_) δ = 8.75 (s, 1H), 8.70 (s, 2H), 7.94 (d, *J* = 8.5 Hz, 2H), 6.98 (d, *J* = 8.5 Hz, 2H), 5.32 (s, 1 H, OH), 4.00 (s, 6H). ^13^C NMR (151 MHz, CDCl_3_) δ = 166.06, 159.13, 153.04, 147.11, 131.87, 131.82, 127.74, 125.66, 116.10, 52.72. HRMS (ESI, positive mode) *m*/*z* calculated for C_16_H_15_N_2_O_5_ [M + H]^+^: 315.0981; found: 315.0983.

*Compound **AB.Me**, (E)-1-(3,5-dimethylphenyl)-2-(4-methoxyphenyl)diazene.* A solution of compound **AB-OH.Me** (0.100 g, 0.442 mmol, 1 eq), iodomethane (0.033 mL, 0.530 mmol, 1.2 eq), K_2_CO_3_ (0.091 g, 0.663 mmol, 1.5 eq), and dimethylformamide (2 mL) was stirred at 7 °C for 24 h. The mixture was allowed to reach room temperature, diluted with ethyl acetate, transferred in a separatory funnel, and washed with water. The organic layers were collected, dried over Na_2_SO_4_, filtered, and concentrated in vacuo. The crude product was purified by silica chromatography (petroleum ether/ethyl acetate = 8:2) to obtain the desired product as a bright orange oil (0.088 g, 83.0%). ^1^H NMR (600 MHz, CDCl_3_) δ 7.91 (d, *J* = 9.0 Hz, 2H), 7.50 (s, 2H), 7.09 (s, 1H), 7.02 (d, *J* = 9.0 Hz, 2H), 3.89 (s, 3H), 2.41 (s, 6H). ^13^C NMR (151 MHz, CDCl_3_) δ 162.06, 153.11, 147.25, 138.81, 132.22, 124.75, 120.48, 114.33, 55.70, 21.40. HRMS (ESI, positive mode) *m*/*z* calculated for C_15_H_16_N_2_O [M + H]^+^: 241.1341; found: 241.1341.

*Compound **AB.OMe**, (E)-1-(3,5-dimethoxyphenyl)-2-(4-methoxyphenyl)diazene*. A solution of compound **AB-OH.OMe** (0.146 g, 0.566 mmol, 1 eq), iodomethane (0.042 mL, 0.680 mmol, 1.2 eq), K_2_CO_3_ (0.117 g, 0.849 mmol, 1.5 eq), and dimethylformamide (2.5 mL) was stirred at 70 °C for 24 h. The mixture was allowed to reach room temperature, diluted with ethyl acetate, transferred in a separatory funnel, and washed with water. The organic layers were collected, dried over Na_2_SO_4_, filtered and concentrated in vacuo. The crude product was purified by silica chromatography (petroleum ether/ethyl acetate = 8:2) to obtain the desired product as a bright orange powder (0.084 g, 54.5%). ^1^H NMR (600 MHz, CDCl_3_) δ 7.92 (d, *J* = 9.4 Hz, 2H), 7.09 (d, 2H), 7.03 (d, *J* = 9.4 Hz, 2H), 6.58 (s, 1H), 3.90 (s, 3H), 3.88 (s, 6H). ^13^C NMR (151 MHz, CDCl_3_) δ 162.32, 161.22, 154.76, 146.99, 124.98, 114.38, 103.49, 100.77, 76.95, 55.74. HRMS (ESI, positive mode) *m*/*z* calculated for C_15_H_16_N_2_O_3_ [M + H]^+^: 273.1239; found: 273.1238.

*Compound **AB.CO_2_Me**, (E)-dimethyl 5-((4-methoxyphenyl)diazenyl)isophthalate*. A solution of compound **AB-OH.CO_2_Me** (0.100 g, 0.318 mmol, 1 eq), iodomethane (0.024 mL, 0.382 mmol, 1.2 eq), K_2_CO_3_ (0.066 g, 0.477 mmol, 1.5 eq) and dimethylformamide (1.5 mL) was stirred at 70 °C for 24 h. The mixture was allowed to reach room temperature, diluted with ethyl acetate, transferred in a separatory funnel, and washed with water. The organic layers were collected, dried over Na_2_SO_4_, filtered, and concentrated in vacuo. The crude product was purified by silica chromatography (dichloromethane 100%) to obtain the desired product as a bright yellow powder (0.033 g, 31.7%). ^1^H NMR (600 MHz, CDCl_3_) δ = 8.75 (s, 1H), 8.70 (s, 1H), 7.98 (d, *J* = 8.7 Hz, 2H), 7.05 (d, *J* = 8.7 Hz, 2H), 3.99 (s, 6H), 3.91 (s, 3H). ^13^C NMR (151 MHz, CDCl_3_) δ = 166.00, 162.90, 153.04, 146.86, 131.75, 127.67, 125.39, 114.48, 55.76, 52.67. HRMS (ESI, positive mode) *m*/*z* calculated for C_17_H_16_N_2_O_5_ [M + H]^+^: 329.1137; found: 329.1130.

## 4. Conclusions

We investigated azobenzene derivatives functionalized at their *meta* position with electron-directing substituents. The spectral and photoswitching changes induced by the presence of electron-donating and electron-withdrawing groups were compared to a reference compound—an azobenzene inclusive of two methyl units at its *meta* positions. The azobenzene functionalized with electron-donating groups (OMe) exhibits modest changes in the absorption spectrum in both *E* and *Z* isomers, closely resembling the spectral signatures of the reference methylated counterpart. In contrast, functionalization with electron-withdrawing groups (CO_2_Me) induced an 11 nm bathochromic shift in the π–π* high-energy band of the *E* isomer and a 21 nm shift in the main π–π* band of its *Z* counterpart. The time of thermal relaxation *Z→E* is limitedly affected by the presence of methoxyl functionality, whereas longer time (over a day more) is needed for the carbonyl groups. Regardless, the τ_1/2_ of the thermal relaxation of three azobenzenes take place within similar time range with ~2 h of difference (6–8 h). Our computational studies confirmed that introducing electron-directing substituents at the *meta* position affects the energy levels of the frontier orbitals of azobenzene and enhances the stability of the *Z* and *E* isomers for the electron-withdrawing carbonyl groups. Our findings enhance our understanding of the structure–property relationship of azobenzene, enabling us to selectively tune its optical properties and thermal relaxation behavior. This newfound knowledge represents a valuable addition to the toolkit for designing functional molecular switches.

## Data Availability

The data presented in this study are available on request from the corresponding author. The data are not publicly available due to specific ethical and privacy considerations.

## Supplementary Materials

The following supporting information can be downloaded at: https://www.mdpi.com/article/10.3390/molecules29091929/s1. All of the ^1^H and ^13^C NMR spectra are available in the Supplementary Materials. Absorption measurements and cyclability test, *E*/*Z* isomerization experiments, thermal relaxation mathematical models, and DFT calculations are explored in more detail in the Supplementary Materials. Scheme S1: Synthetic scheme for the preparation of the *meta*-substituted azobenzenes. Table S1: Irradiation time and sample concentration for **AB.Me, AB.OMe**, and **AB.CO_2_Me**. Table S2: Cyclability: irradiation time for **AB.Me**, **AB.OMe**, and **AB.CO_2_Me**. Tables S3 and S4: Thermal relaxation parameters and fitting results (Figure S8, logistic modeland logarithmic model). Table S5: Total energy of azobenzene derivatives in *E* and *Z* configurations. Table S6: Bandgap (Eg) and energy level of HOMO and LUMO states of azobenzene derivatives in *E* (a) and *Z* (b) configuration. Zero energy reference is set to the vacuum level. Figure S1 aromatic region of the ^1^H-NMR spectrum of compound **AB.Me** (a), **AB.OMe** (b), **AB.CO_2_Me** (c) in chloroform-d. Figures S2, S4, S6, panoramic ^1^H-NMR spectra of *E* form (a), *Z* form (b), and PSS state (c) for **AB.Me**, **AB.OMe**, and **AB.CO_2_Me** in chloroform-d. Figures S3, S5, S7, panoramic ^1^H-NMR spectra of *E* form (a), *Z* form (b), and PSS state (c) for **AB.Me**, **AB.OMe**, and **AB.CO_2_Me** in THF-d8. Figure S8: Experimental data points of thermal relaxation for **AB.Me**, **AB.OMe**, and **AB.CO_2_Me**. Figure S9: Density of states (DOS) of azobenzene derivatives in *E* (a) and *Z* (b) configuration. Zero energy reference is set to HOMO state of each system. References [41,42,43,44,45,46] are citation in the Supplementary Materials.
