# Supplementary material for: Meta-Substituted Asymmetric Azobenzenes: Insights into Structure–Property Relationship
Source: Molecules. 2024 Apr 23;29(9):1929. doi: 10.3390/molecules29091929 (PMC11085191; doi:10.3390/molecules29091929)
Supplement: Supplementary file 1 [file molecules-29-01929-s001.zip › molecules-2973186-supplementary-proof.pdf]

Supplementary Materials

Meta-Substituted Asymmetric Azobenzenes: Insights Into Structure-Property Relationship

Anna Laura Sanna[1], Tatiana Pachova[2], Alessandra Catellani[3], Arrigo Calzolari[3,*] and Giuseppe Sforazzini[1,2,*]

[1]*Department of Chemical and Geological Sciences, Università degli Studi di Cagliari, 09042 Monserrato, Cagliari, Italy. email: giuseppe.sforazzini@unica.it*

[2]*Laboratory of Macromolecular and Organic Materials, Institute of Material Science and Engineering, Ecole Polytechnique Federale de Lausanne (EPFL), 1015 Lausanne, Switzerland.*

[3]*CNR-NANO, Istituto Nanoscienze,* Via *Giuseppe Campi, 213, 41125 Modena, Italy* email: *arrigo.calzolari@nano.cnr.it*

Table of contents

1. Synthetic Details 2

1.1. Scheme for Azobenzenes Preparation 2

1.2. ^{1}H NMR Spectra 3

1.3. ^{13}C NMR Spectra 6

2. Isomerization and PSS experiments for AB.Me, AB.OMe, AB.CO$_2$Me 9

2.1. ^{1}H NMR spectra of *E*/*Z* isomerization and PSS of AB.Me 11

2.2. ^{1}H NMR spectra of *E*/*Z* isomerization and PSS of AB.OMe 13

2.3. ^{1}H NMR spectra of *E*/*Z* isomerization and PSS of AB.CO$_2$Me 15

3. Absorption Spectra and Cyclability 17

4. Thermal relaxation 18

4.1. Logistic Model 19

4.2. Logarithmic Model 20

4.3. Fitting performance 21

5. DFT simulations 21

5.1 Computational details 21

5.2 DFT analysis 21

6. References 23

1. Synthetic Details

1.1. Scheme for Azobenzenes Preparation

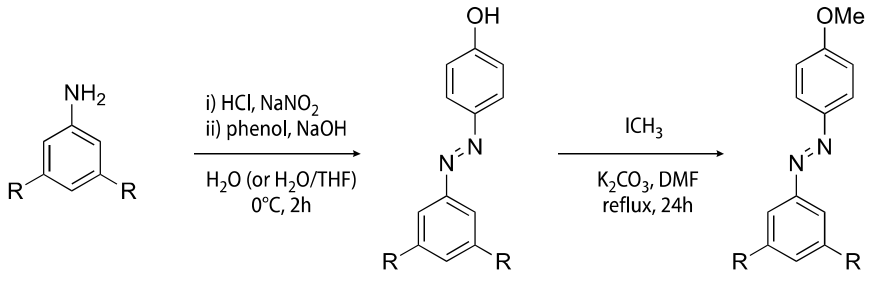

Ph-NH$_2$.Me ; R = Me
Ph-NH$_2$.OMe ; R = OMe
Ph-NH$_2$.CO$_2$Me ; R = CO$_2$Me

AB-OH.Me ; R = Me
AB-OH.OMe ; R = OMe
AB-OH.CO$_2$Me ; R = CO$_2$Me

AB.Me ; R = Me
AB.OMe ; R = OMe
AB.CO$_2$Me ; R = CO$_2$Me

Scheme S1. Synthetic scheme for the preparation of the *meta*-substituted azobenzenes.

1.2. ^{1}H NMR Spectra

Compound AB-OH.Me

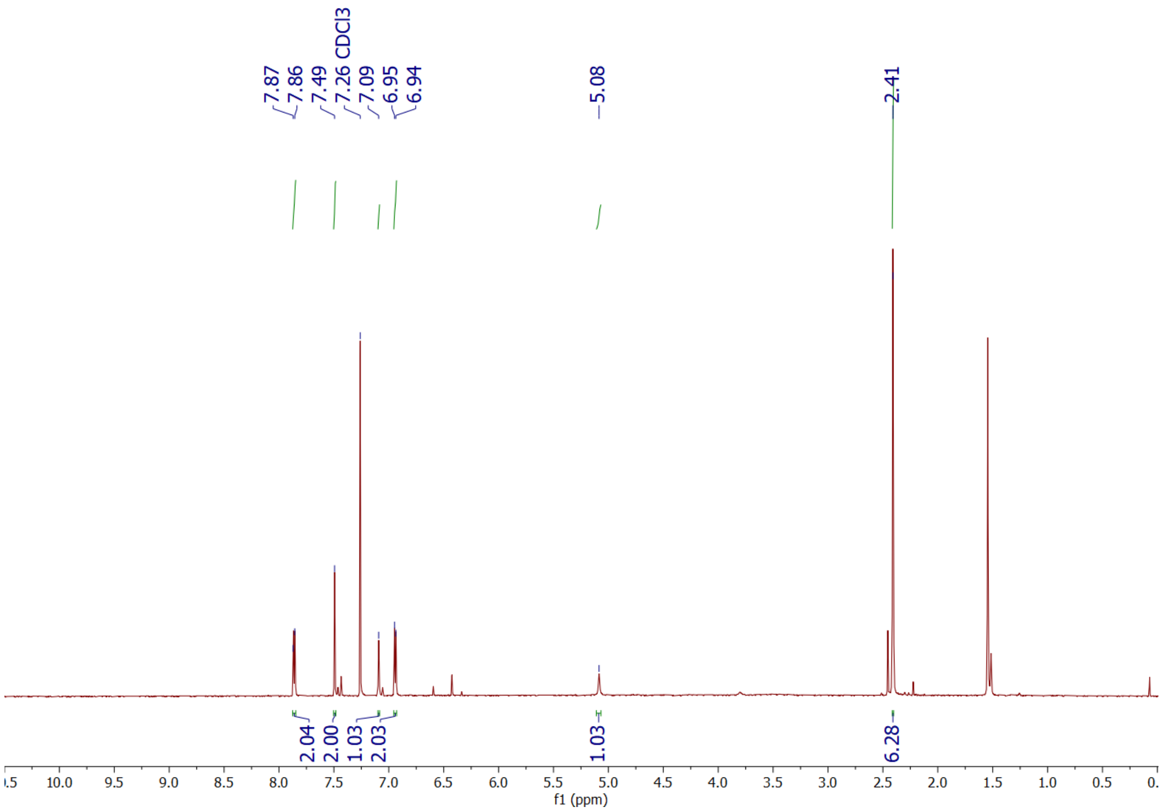

Compound AB-OH.OMe

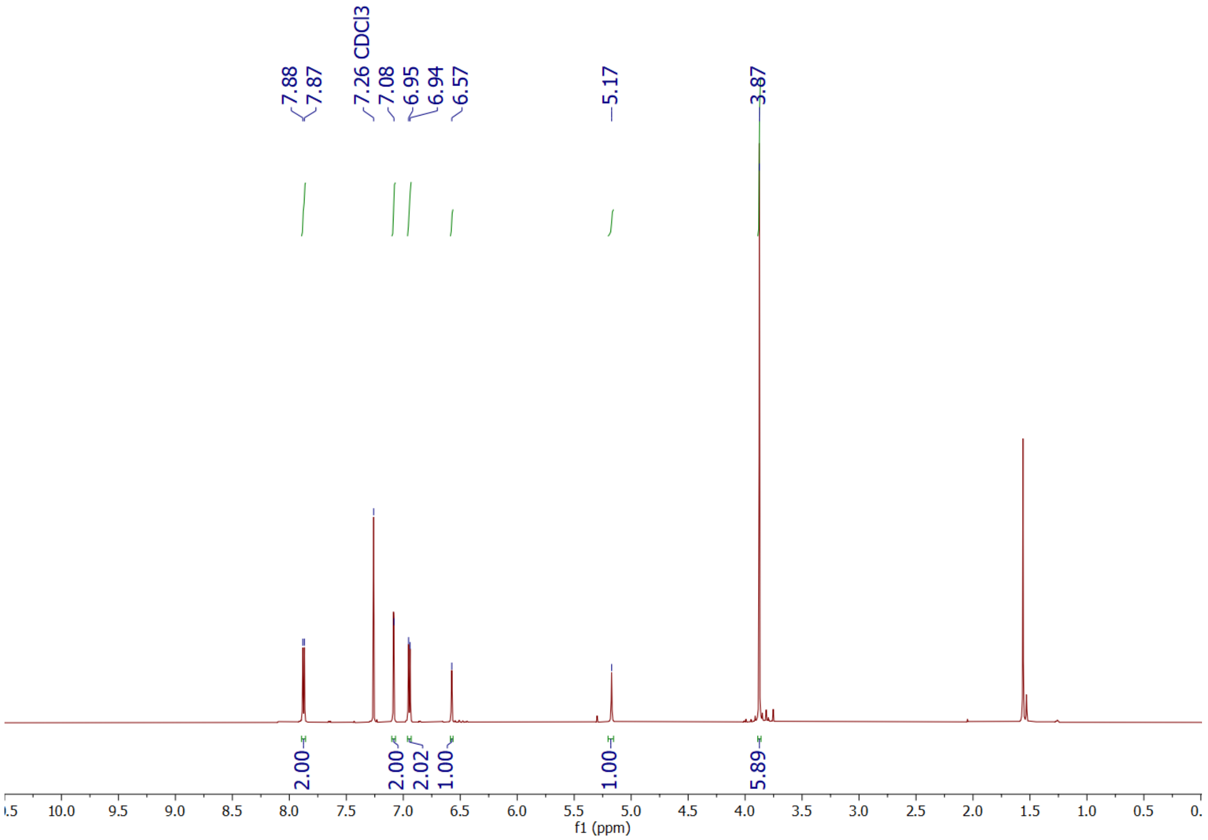

Compound AB-OH.CO_2Me

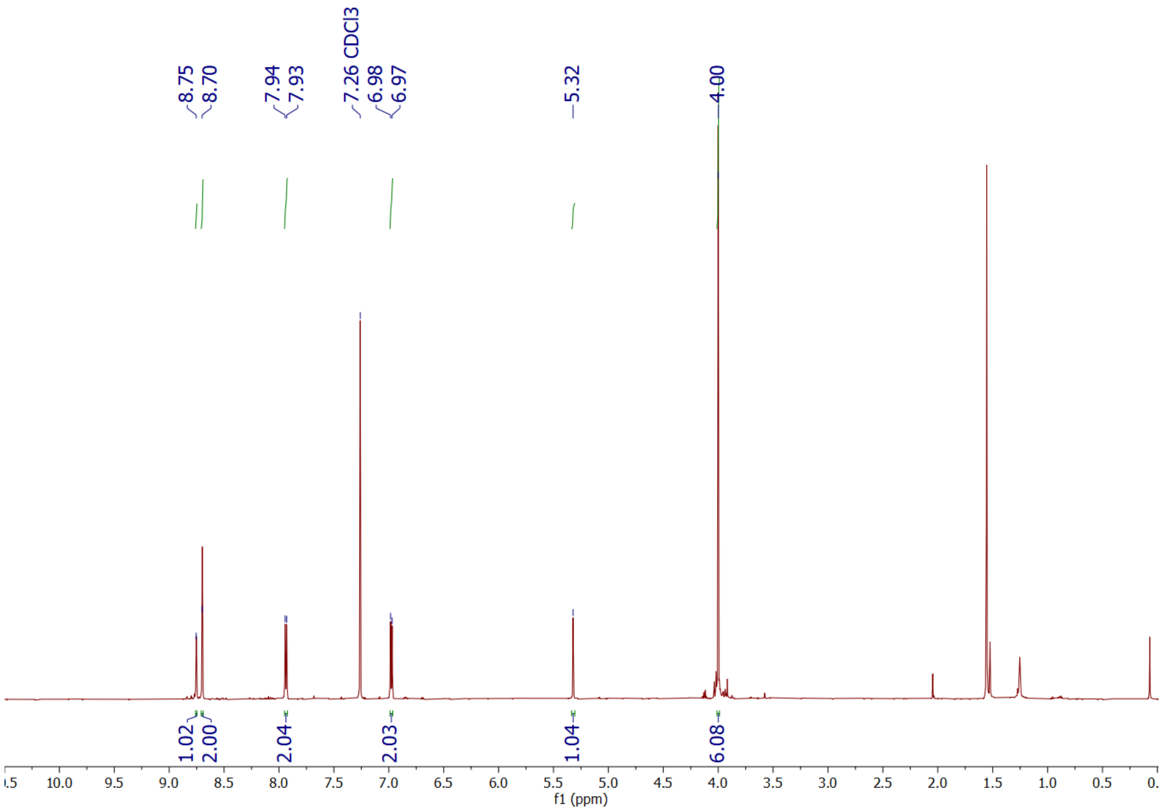

Compound AB.Me

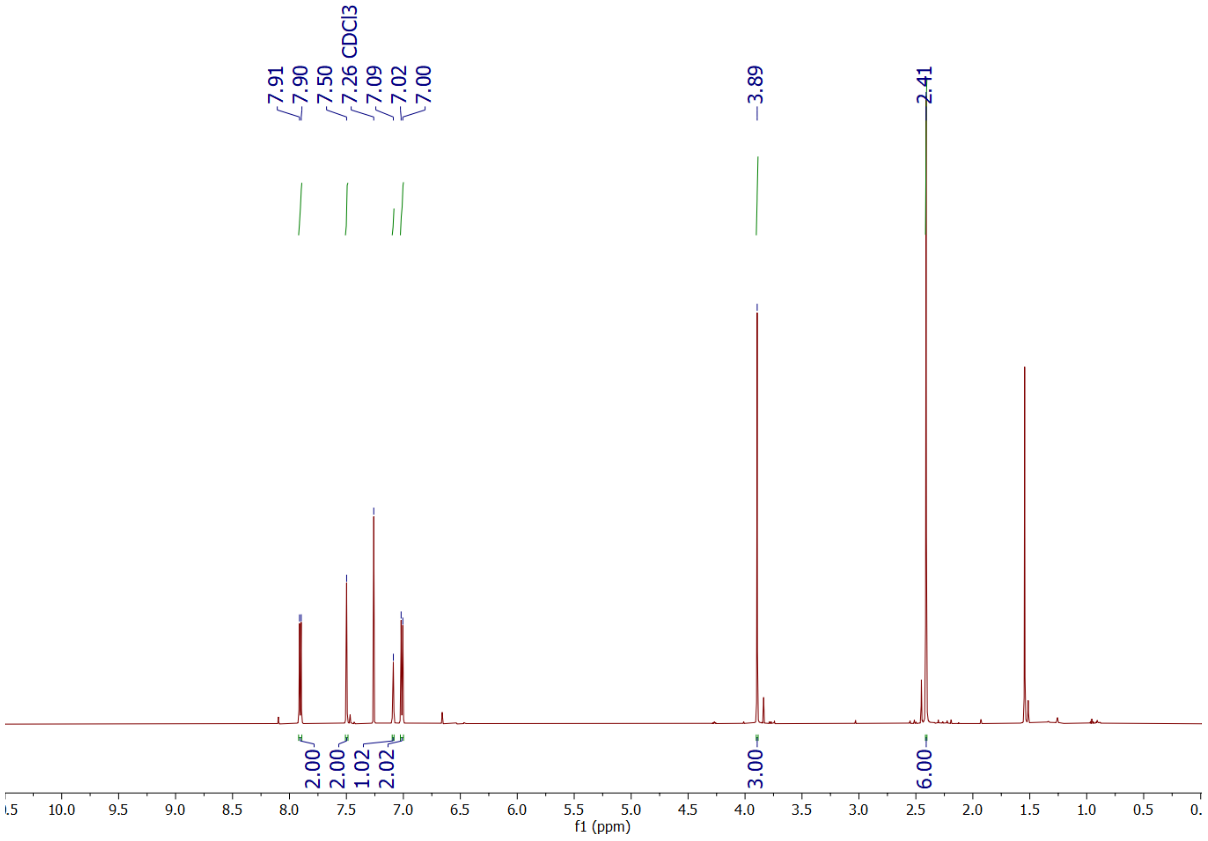

Compound AB.OMe

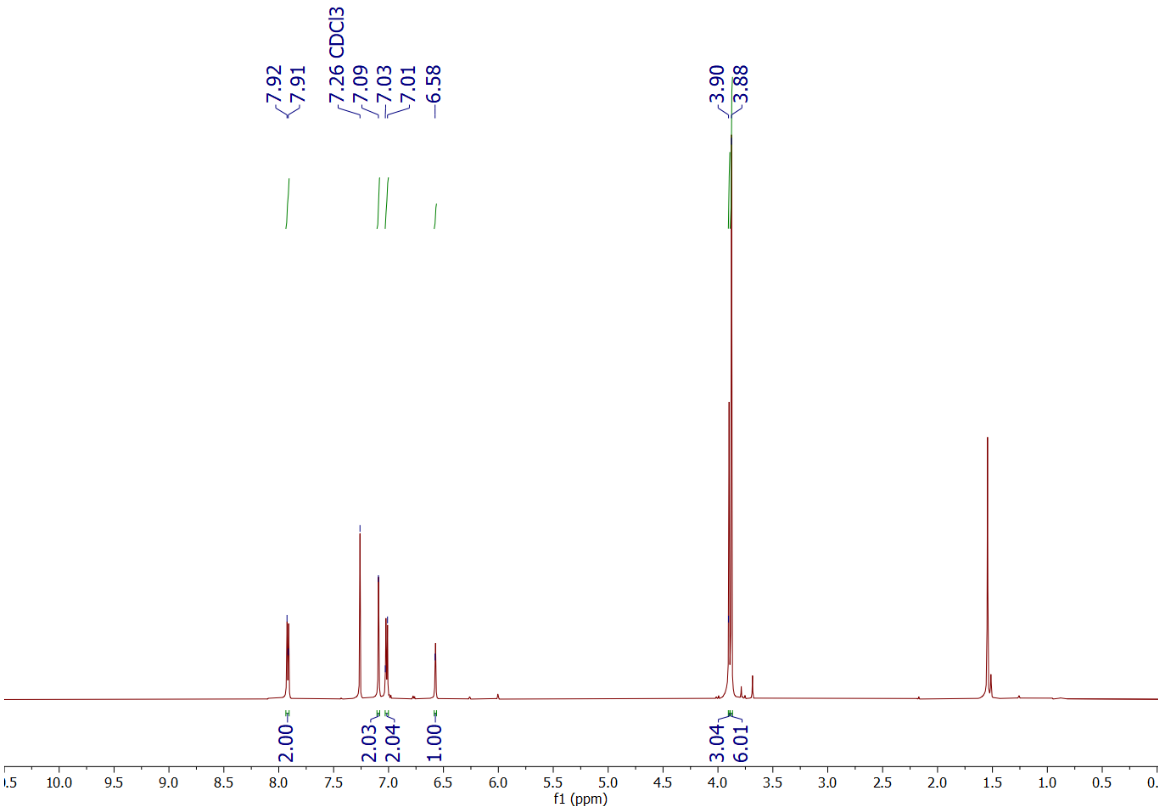

Compound AB.CO$_2$Me

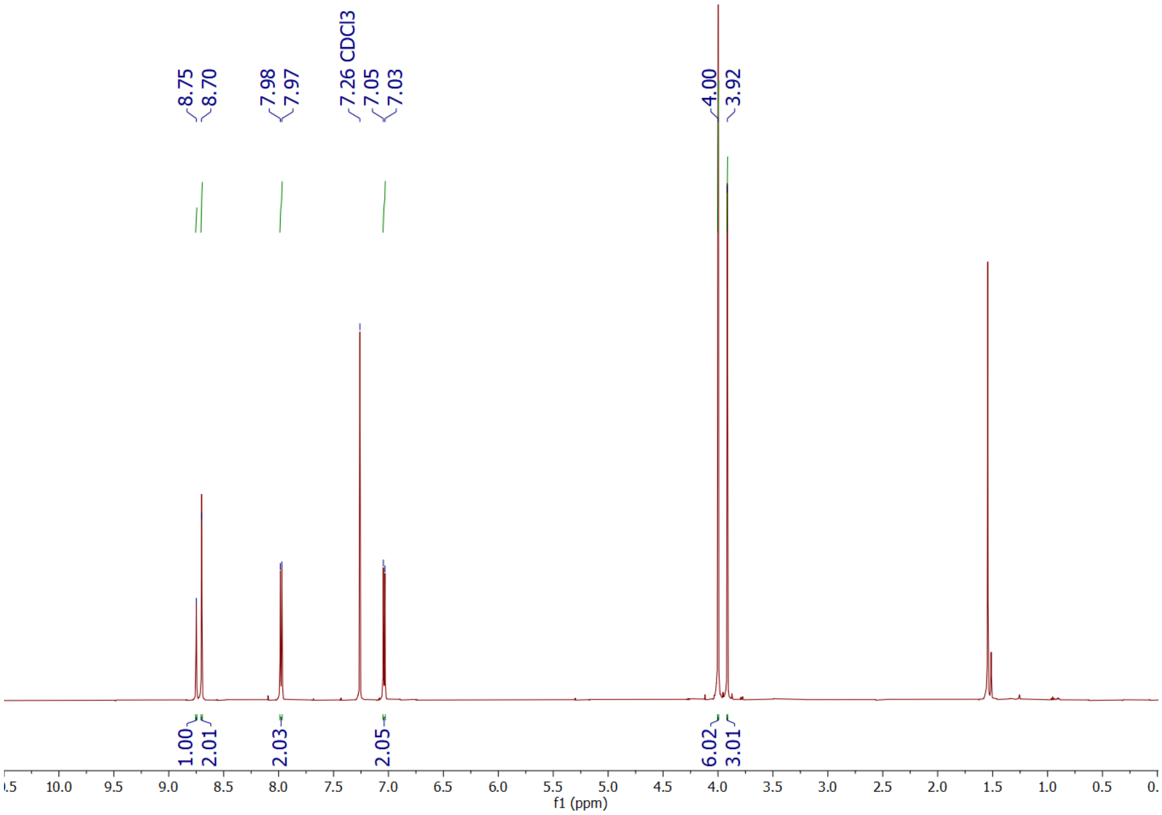

1.3. ^13C NMR Spectra

Compound AB-OH.Me

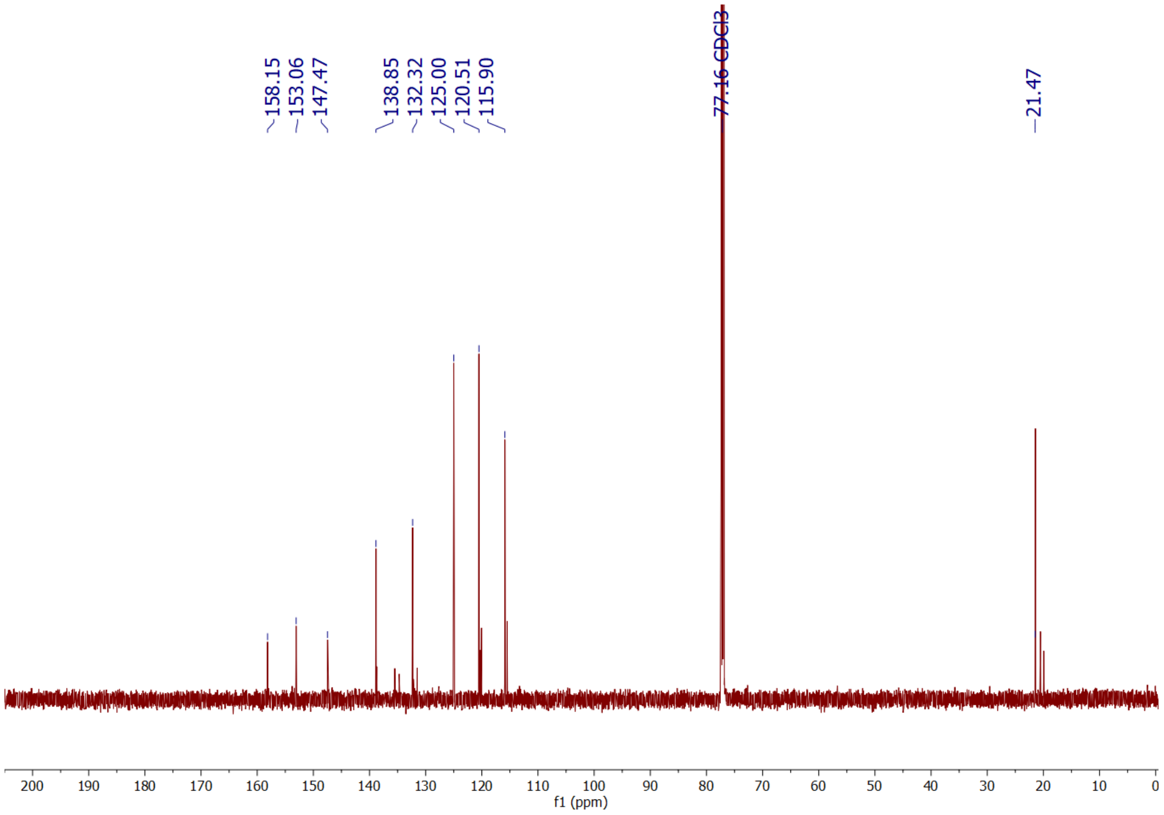

Compound AB-OH.OMe

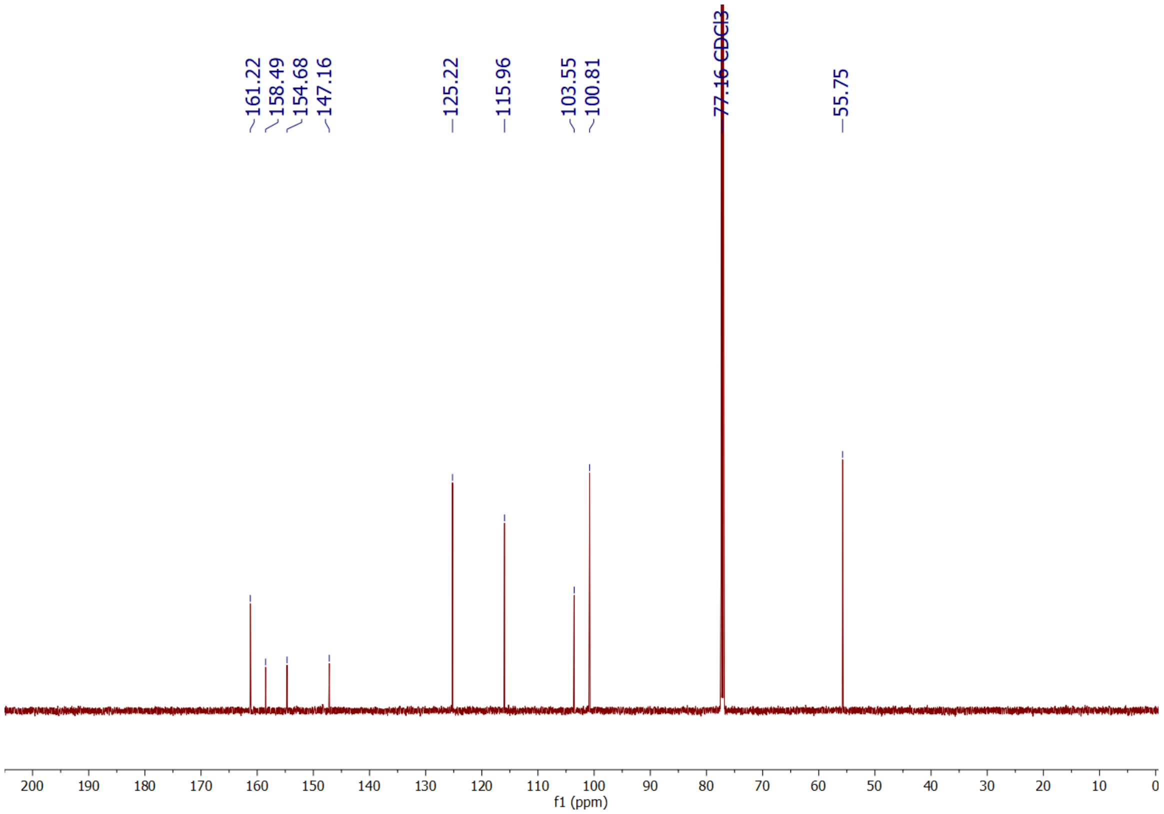

Compound AB-OH.CO_2Me

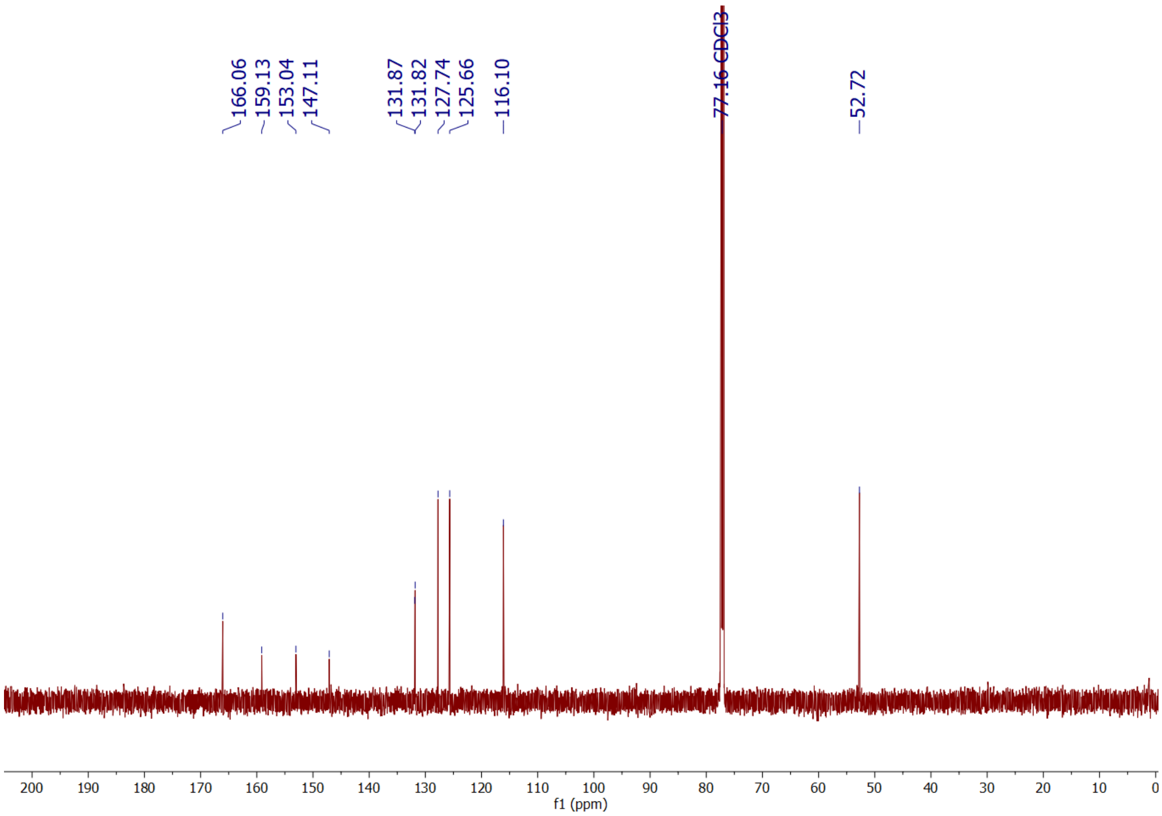

Compound AB.Me

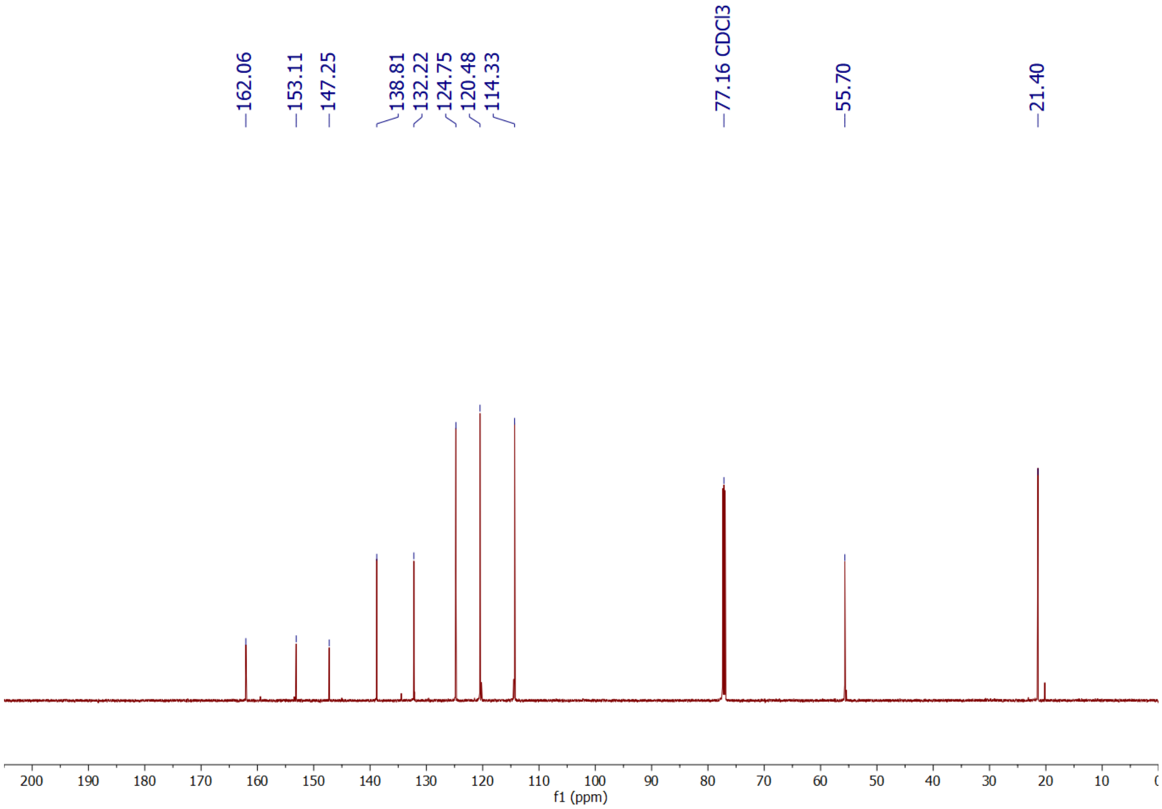

Compound AB.OMe

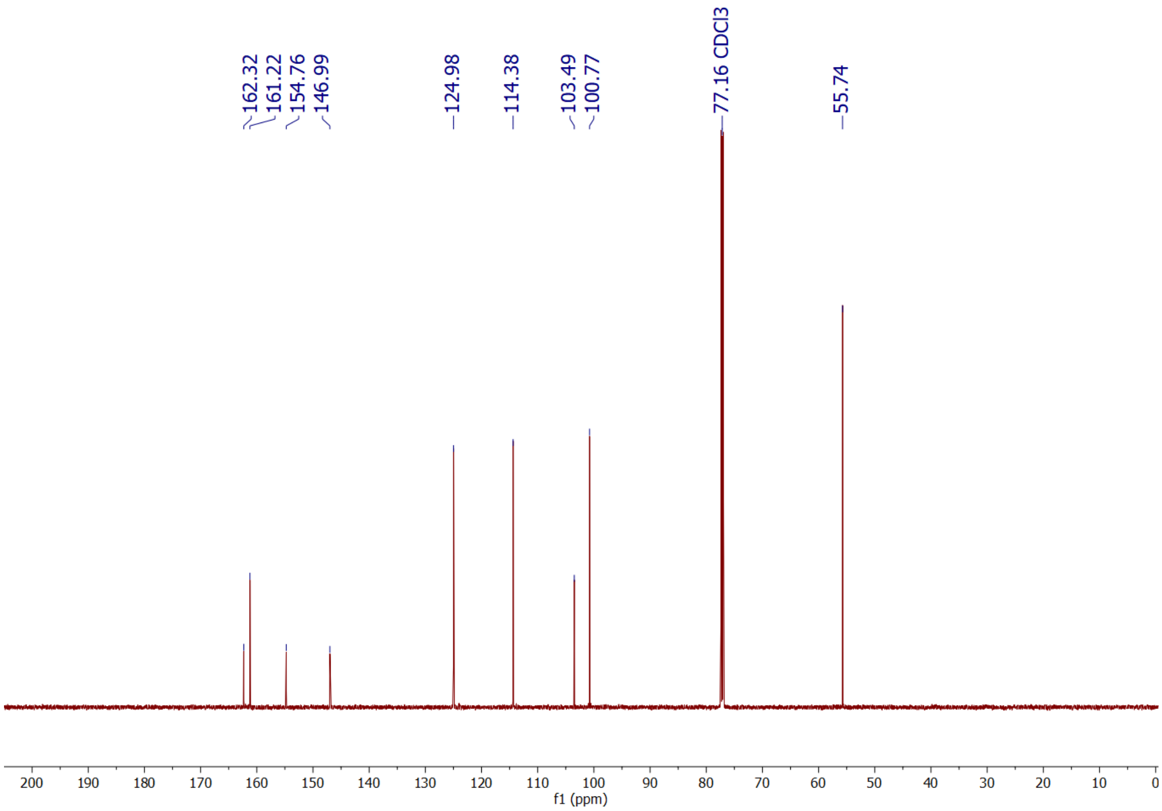

Compound AB.CO_2Me

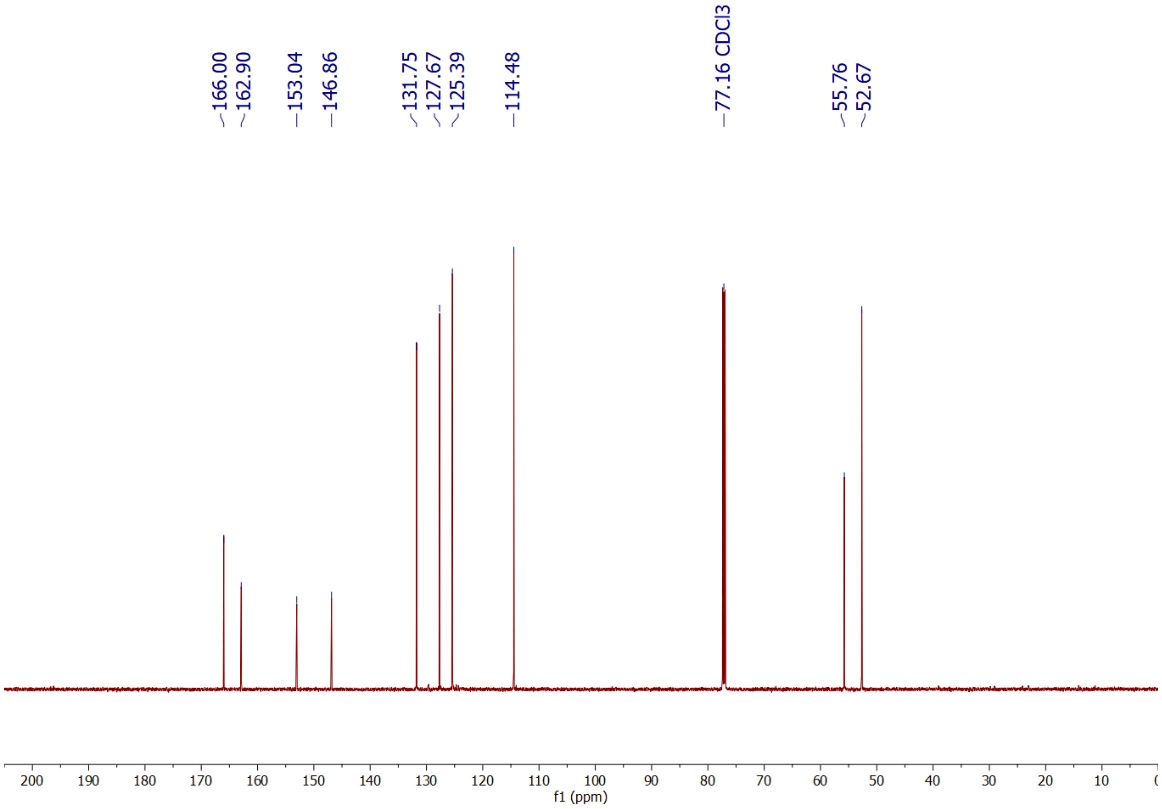

2. Isomerization and PSS experiments for AB.Me, AB.OMe, AB.CO_2Me

Isomerization experiments from *E* to *Z* were performed on Compound **AB.Me**, **AB.OMe** and **AB.CO_2Me** and PSS conditions were determined through ^{1}H-NMR spectroscopy. Samples of **AB.Me**, **AB.OMe** and **AB.CO_2Me** were prepared with the same quantity (0.00415 mmol) of product in $CDCl_3$. Samples were irradiated at 25°C with 365 nm wavelength for t_{365nm} to promote the *Z* form of compounds and ^{1}H-NMR spectra acquired. Then, the samples were irradiated at 25 °C at 450 nm for t_{450nm} to investigate the PSS conditions and ^{1}H-NMR spectra acquired. Comparison between the *E* form (first spectrum), the *Z* form (second spectrum) and PSS (third spectrum) are shown from Figure S1 to Figure S7. The lamp power used to irradiate at 365 nm is 6 W and the NMR tube was placed at a distance of ~4 cm from the lamp. The lamp used at 450 nm is 1 mW cm^{-2} and the NMR tube was placed at a distance of ~4 cm.

Table S1. Irradiation time and sample concentration for **AB.Me**, **AB.OMe** and **AB.CO_2Me.**

Compound	t_{365nm} [min]	t_{450nm}[min]	Solvent
**AB.Me**	5	225	$CDCl_3$
**AB.OMe**	50	200	
**AB.CO_2Me**	60	60	
**AB.Me**	40	170	THF-d_8
**AB.OMe**	40	170	
**AB.CO_2Me**	40	170	

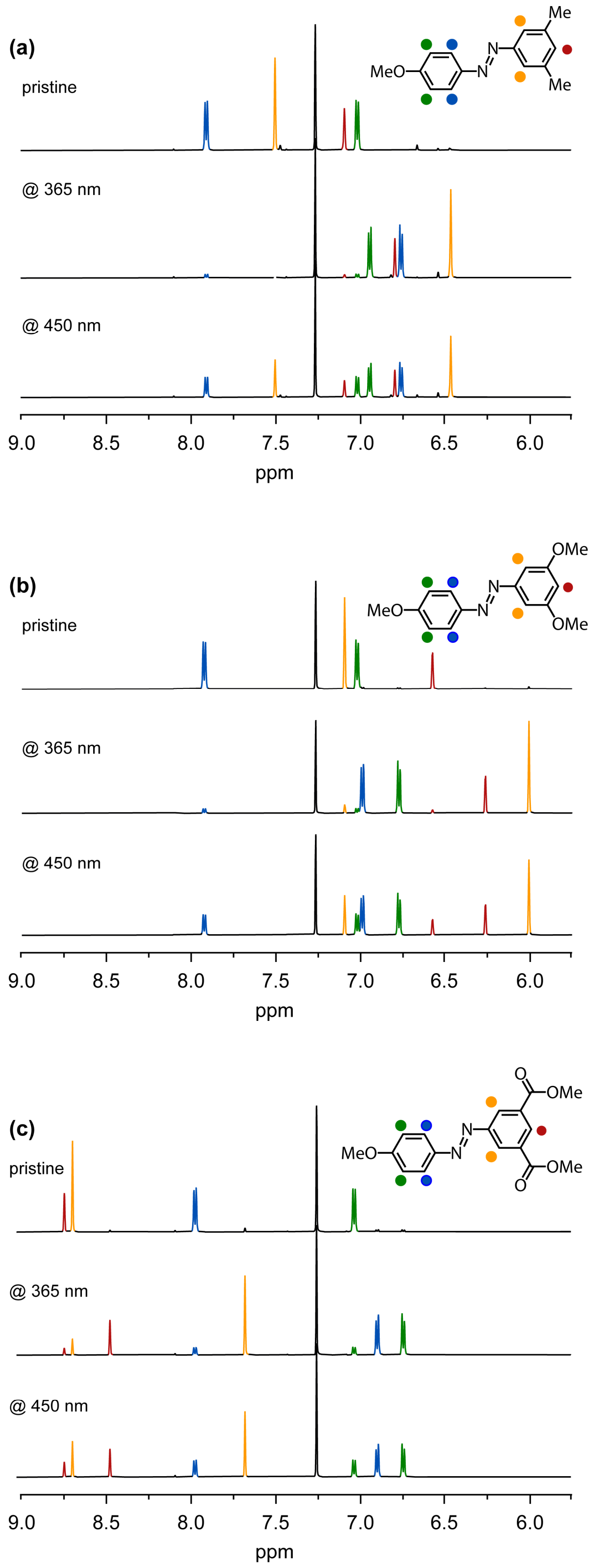

Figure S1. Aromatic region of the ^{1}H-NMR spectrum of compound **AB.Me** (a), **AB.OMe** (b), **AB.CO$_2$Me** (c) in chloroform-d. Pristine (top spectrum), after irradiation at 365 nm (middle spectrum), and after irradiation at 450 nm (bottom spectrum).

2.1. ¹H NMR spectra of *E*/*Z* isomerization and PSS of AB.Me

¹H-NMR ($CDCl_3$) spectroscopy shows that when **AB.Me.*E*** is exposed to 365 nm light, ~90% of **AB.Me.*E*** is converted to **AB.Me.*Z***. By irradiating at 450 nm, the inverted process occurs obtaining ~41% of **AB.Me.*E*** and ~59% of **AB.Me.*Z***.

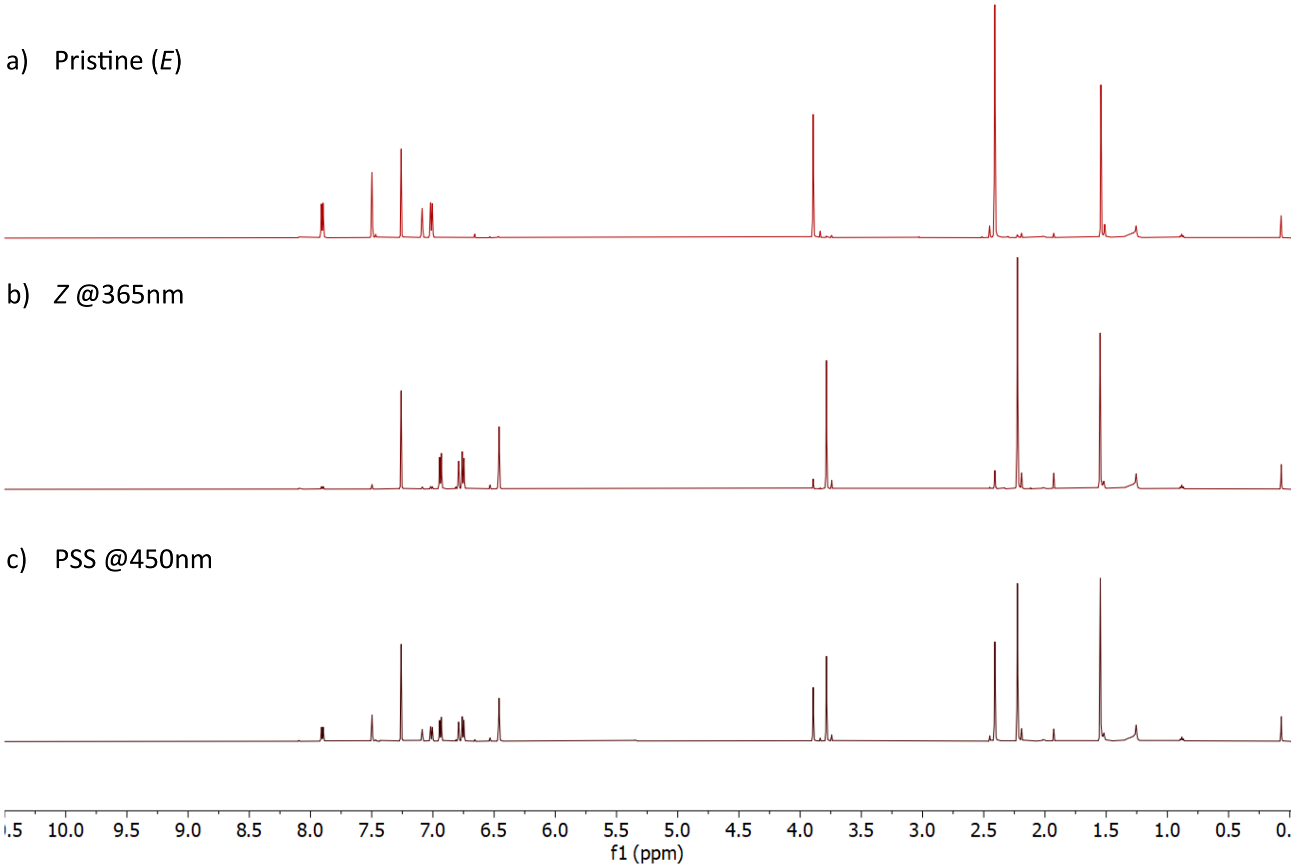

Figure S2. *E* form (a), *Z* form (b) and PSS state (c) for **AB.Me** ($CDCl_3$).

^1H-NMR (THF-d_8) spectroscopy shows that when **AB.Me.*E*** is exposed to 365 nm light, ~93% of **AB.Me.*E*** is converted to **AB.Me.*Z***. By irradiating at 450 nm, the inverted process occurs obtaining ~51% of **AB.Me.*E*** and ~49% of **AB.Me.*Z***.

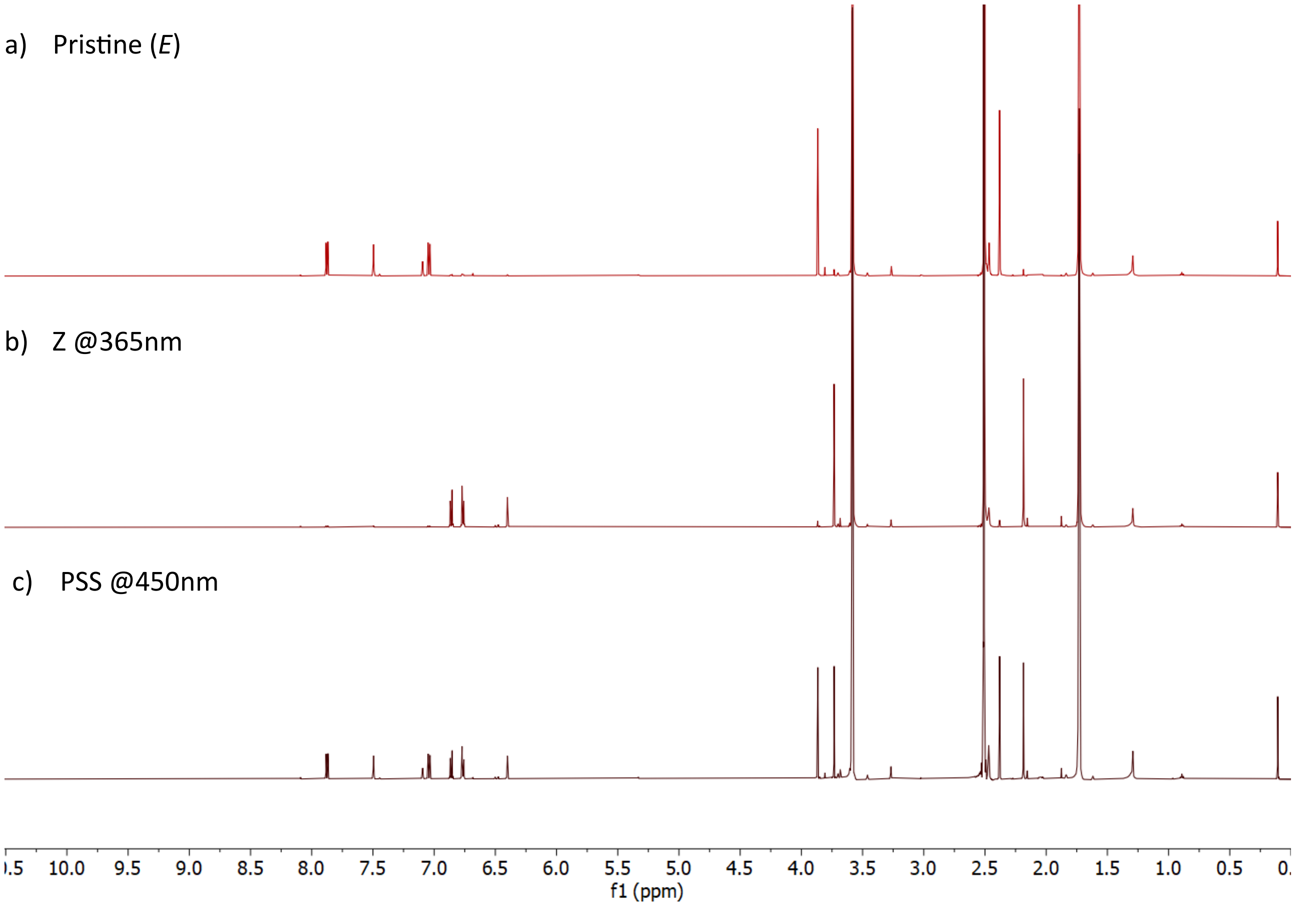

Figure S3. E form (a), Z form (b) and PSS state (c) for AB.Me (THF-d_8).

2.2. ^{1}H NMR spectra of *E*/*Z* isomerization and PSS of AB.OMe

^{1}H-NMR ($CDCl_3$) spectroscopy shows that when **AB.OMe.*E*** is exposed to 365 nm light, ~88% of **AB.OMe.*E*** is converted to **AB.OMe.*Z***. By irradiating at 450 nm, the inverted process occurs obtaining ~34% of **AB.OMe.*E*** and ~66% of **AB.OMe.*Z***.

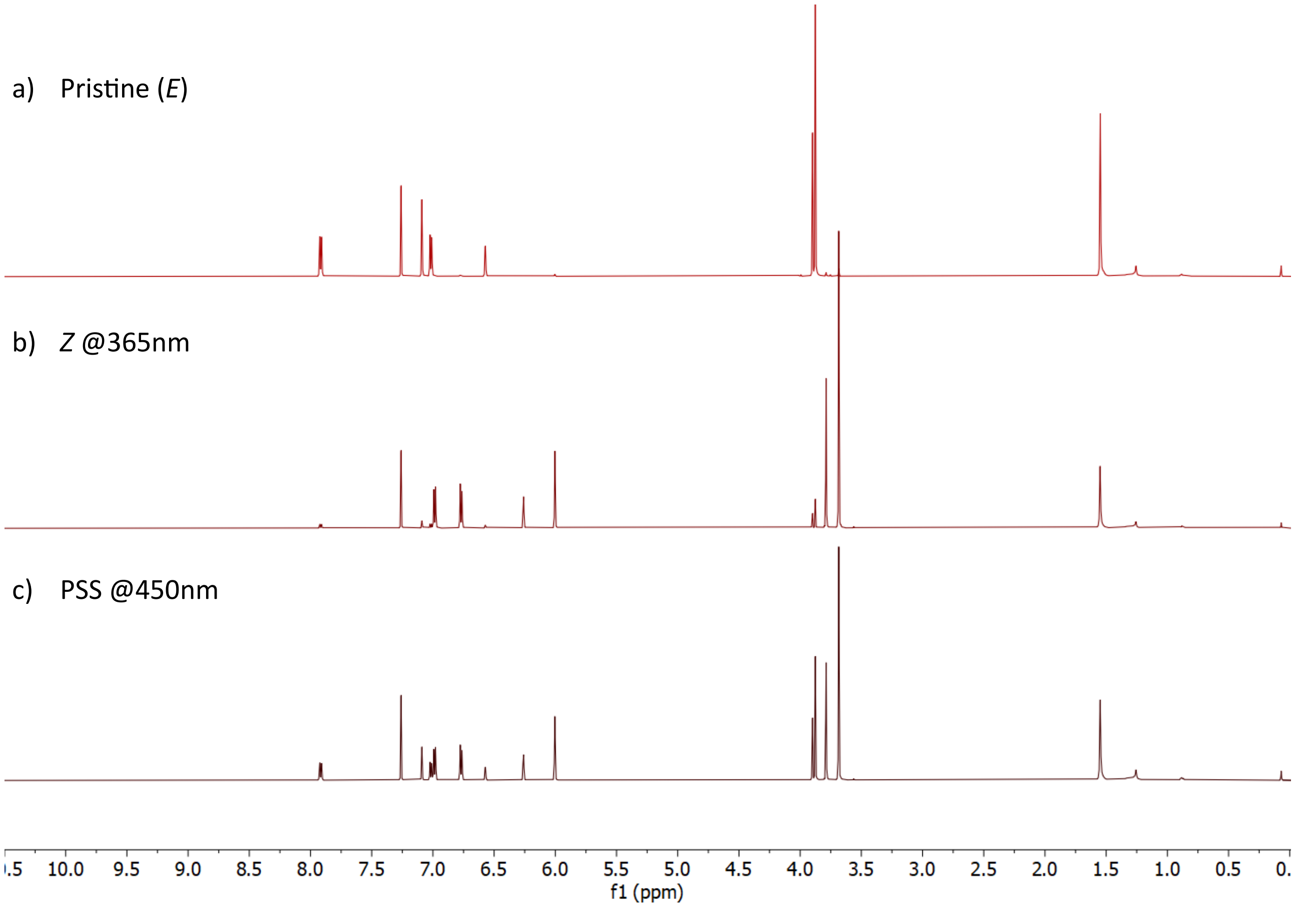

Figure S4. *E* form (a), *Z* form (b) and PSS state (c) for **AB.OMe** ($CDCl_3$).

^{1}H-NMR (THF-d_8) spectroscopy shows that when **AB.OMe.*E*** is exposed to 365 nm light, ~90% of **AB.OMe.*E*** is converted to **AB.OMe.*Z***. By irradiating at 450 nm, the inverted process occurs obtaining ~49% of **AB.OMe.*E*** and ~51% of **AB.OMe.*Z***.

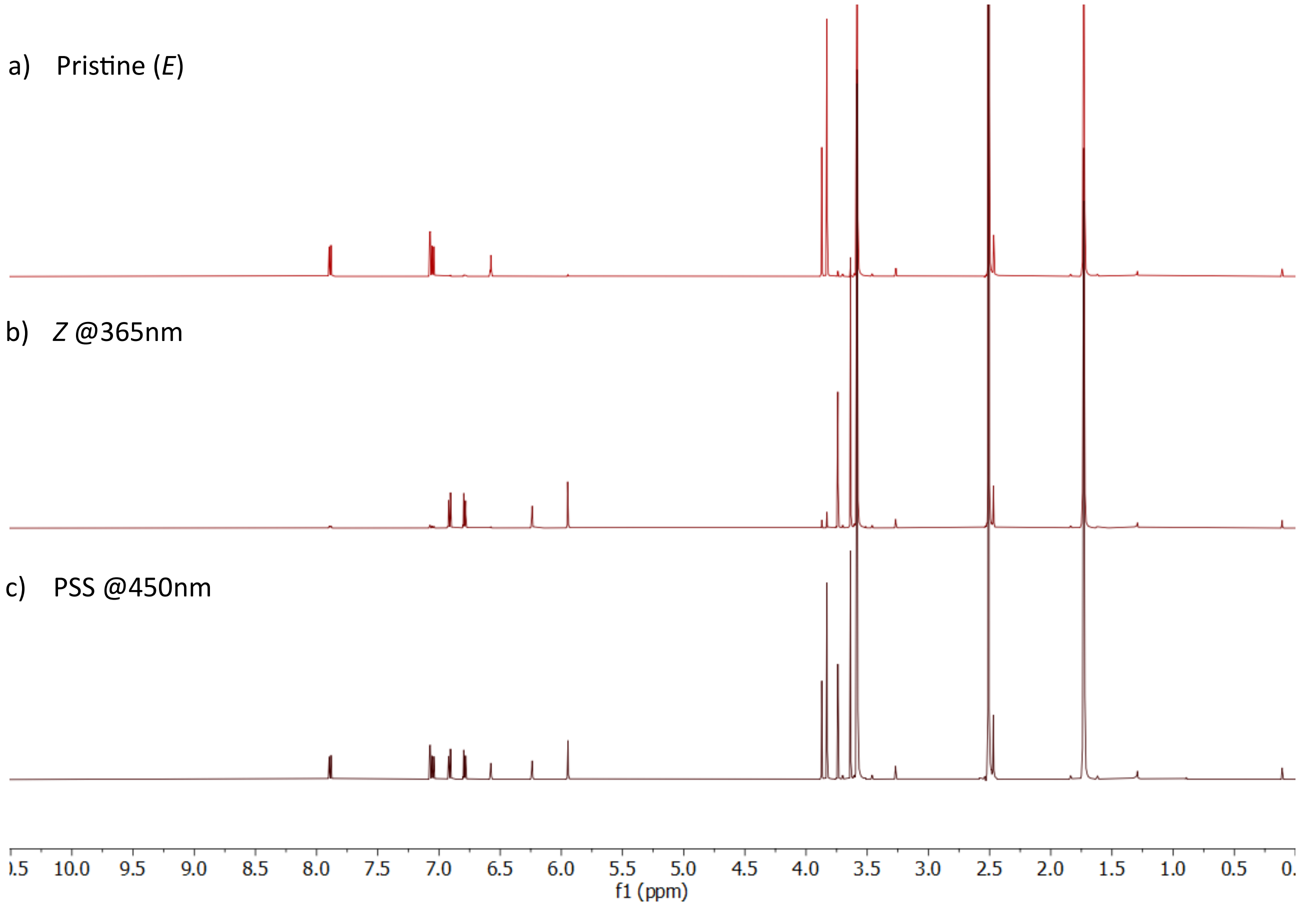

Figure S5. E form (a), Z orm (b) and PSS state (c) for AB.OMe (THF-d_8).

2.3. ^{1}H NMR spectra of *E*/*Z* isomerization and PSS of AB.CO2Me

^{1}H-NMR ($CDCl_3$) spectroscopy shows that when **AB.CO2Me.*E*** is exposed to 365 nm light, ~79% of **AB.CO2Me.*E*** is converted to **AB.CO2Me.*Z***. By irradiating at 450 nm, the inverted process occurs obtaining ~37% of **AB.CO2Me.*E*** and ~63% of **AB.CO2Me.*Z***.

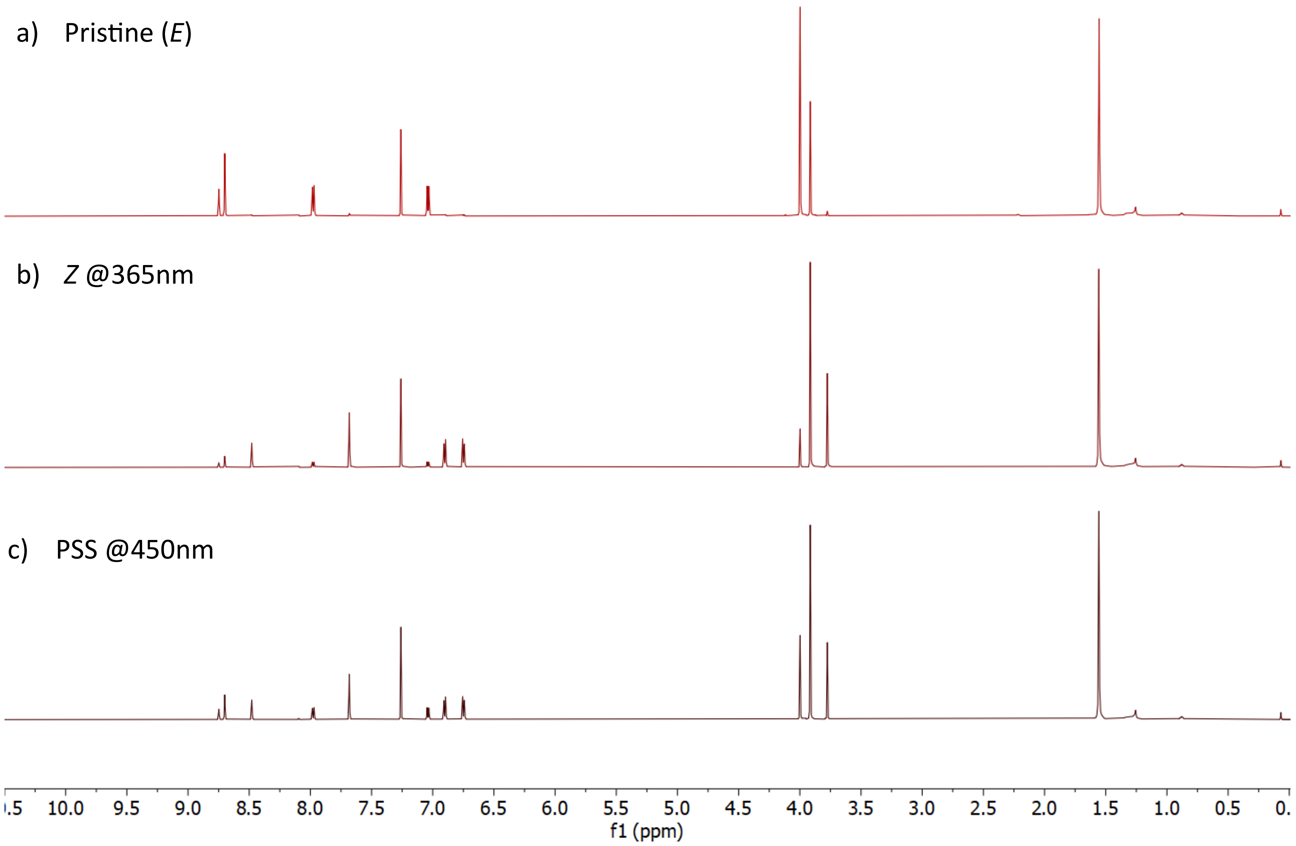

Figure S6. *E* form (a), *Z* form (b) and PSS state (c) for **AB.CO2Me** ($CDCl_3$).

^{1}H-NMR (THF-d$_8$) spectroscopy shows that when **AB.CO$_2$Me.** ***E*** is exposed to 365 nm light, ~89% of **AB.CO$_2$Me.*E*** is converted to **AB.CO$_2$Me.*Z***. By irradiating at 450 nm, the inverted process occurs obtaining ~51% of **AB.CO$_2$Me.*E*** and ~49% of **AB.CO$_2$Me.*Z***.

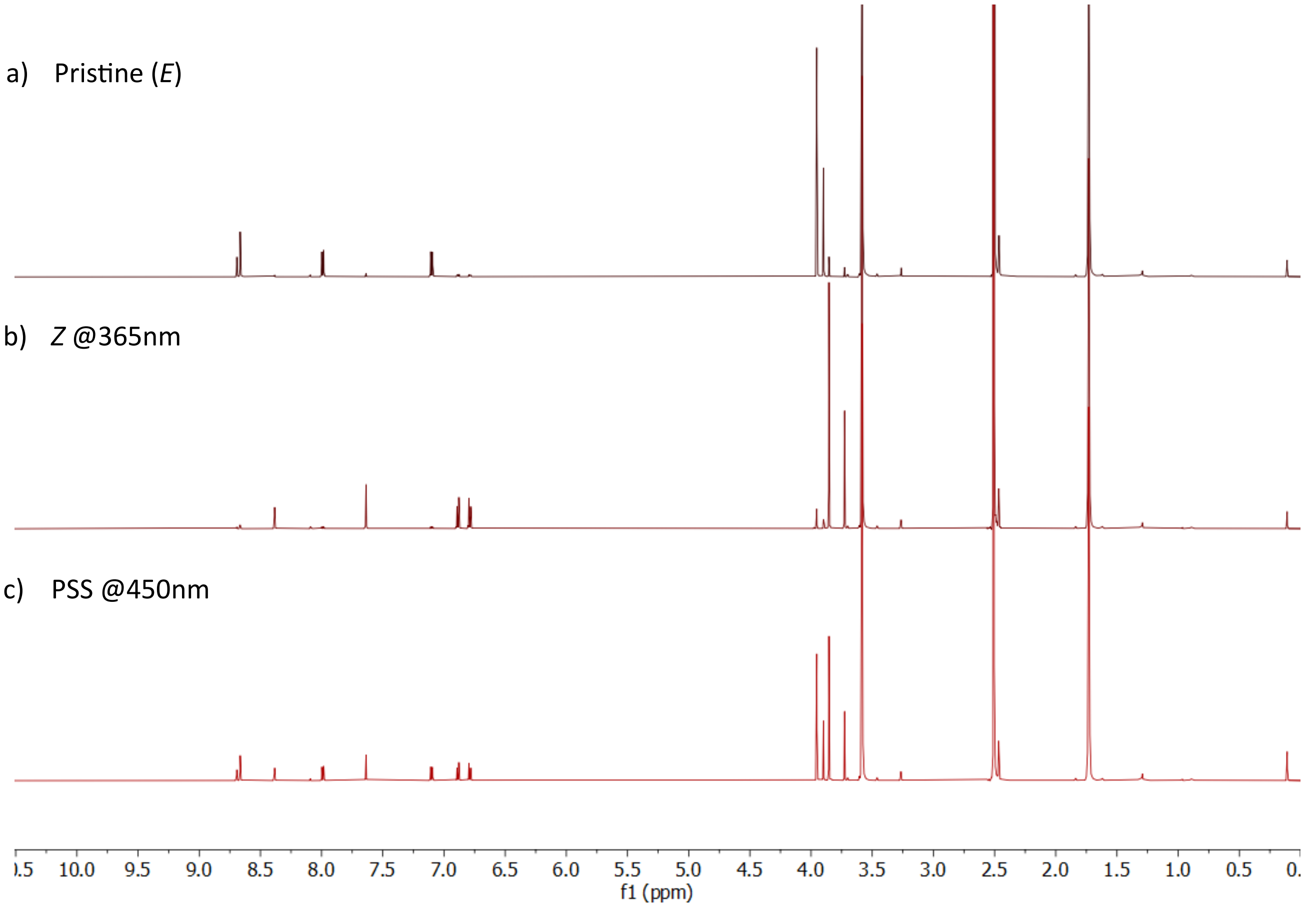

Figure S7. *E* form (a), *Z* form (b) and PSS state (c) for AB.CO$_2$Me (THF-d$_8$).

3. Absorption Spectra and Cyclability

A dilute solutions of **AB.Me, AB.OMe** (~7 x 10^{-6} M) and **AB.CO_2Me** (~2 x 10^{-5} M) in THF were placed ~4 cm from the light source and irradiated with repeated alternating cycles of t_{365nm} with 350 nm (lamp power source 6 W) and t_{450nm} with 450 nm wavelengths nm (lamp source 1 mW cm^{-2}). Irradiation time are reported in Table S2.

Table S2. Cyclability: irradiation time for **AB.Me**, **AB.OMe** and **AB.CO_2Me.**

Compound	t_{365nm} [min]	t_{450nm}[min]
**AB.Me**	13	60
**AB.OMe**	8	30
**AB.COOMe**	10	25

UV-Vis absorption spectra were recorded at 25 °C. Cyclability graphs shown in Figure 2 d-f (in the Article text) are obtained plotting the absorbance value corresponding to the λ_{max} of each compound (350 nm for **AB.Me**, 353 for **AB.OMe** and 358 nm for **AB.CO_2Me**) for each cycle of irradiation.

4. Thermal relaxation

The thermal relaxation kinetics of compounds **AB.Me**, **AB.OMe**, **AB.CO₂Me** were investigated by UV-Vis spectroscopy. Diluted solutions (~1 x 10^{-4} M) of the compounds in THF were prepared and the UV-Vis absorption spectra were recorded at 25 °C. Samples were irradiated with 365 nm wavelength for 30 s and then analysed through UV-vis spectrometer in every 3 hours. Kinetic curves were plotted by following the maximum absorbance of light (at 350 nm for **AB.Me**, 353 nm for **AB.OMe** and 357 nm for **AB.CO₂Me**) during thermal relaxation from *Z* form to the *E* one. Experimental data points of absorbance, $A(t)$, are shown in Figure S4. In order to best fit experimental data points and evaluate the parameters useful to describe the relaxation processes, two kinetics model were taking into account: logarithmic and logistic models.

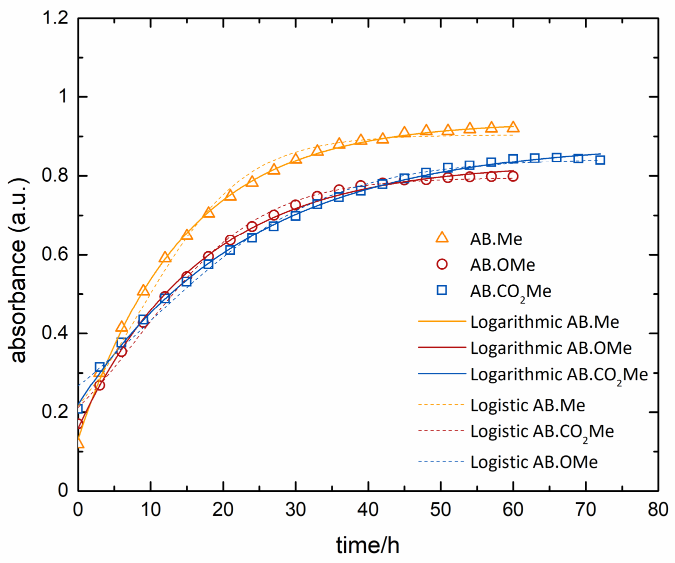

Figure S8. Experimental data points of thermal relaxation for **AB.CO₂Me** (□), **AB.OMe** (○), **AB.Me** (△). Best-fitted lines are shown for Logistic model (dashed lines) and Logarithmic one (continuous lines).

4.1. Logistic Model

The logistic model consist in a sigmoidal curve – logistic type [S1], which equation is:

$$A(t) = \frac{A_\infty}{1+e^{-k_R(t-\tau_{1/2})}} \quad (1)$$

where A_∞ is the maximum amplitude; $\tau_{1/2}$ is the half-life time - the time at which half of the maximum amplitude is reached; k_R is the coefficient of logistic growth rate.

As can be seen from the Figure 1, the logistic curve (dashed lines) overlaps nicely with the experimental points, allowing an accurate estimation of the model quantities A_∞, k_R and $\tau_{1/2}$.

The model parameters are summarized in Table S3.

Table S3. Thermal relaxation parameters and fitting results.

Compound	Parameter	Value	St. Error	Fitting Results	Value
**AB.Me**	A_∞	0.90419	0.01065	**R²**<br>**RSS**	0.98225<br>0.01682
	$\tau_{1/2}$	8.41098 h	0.47022		
	k_R	0.13805 h^{-1}	0.0098		
**AB.OMe**	A_∞	0.79548	0.00533	**R²**<br>**RSS**	0.99491<br>0.00339
	$\tau_{1/2}$	8.60355 h	0.2737		
	k_R	0.11813 h^{-1}	0.0044		
**AB.CO_2Me**	A_∞	0.8449	0.00707	**R²**<br>**RSS**	0.99184<br>0.00624
	$\tau_{1/2}$	9.45788 h	0.41801		
	k_R	0.08169 h^{-1}	0.00356		

4.2. Logarithmic Model

The thermal relaxation can be also described through a logarithmic model [S2], which equation is:

$$\ln \frac{A_0 - A_\infty}{A(t) - A_\infty} = k_R^* t \quad (2)$$

where A_0, $A(t)$, A_∞^* are the Z form absorbances corresponding to the time 0, t and photostationary state; t is the relaxation time and k_R^* is the rate constant of the Z/E relaxation.

Expressing Eq. 2 in relation to the absorbance value we obtain:

$$A(\mathrm{t}) = A_\infty^* + \frac{A_0 - A_\infty}{e^{k_R^* t}} \quad (3)$$

As can be seen from the Figure 1, the logarithmic curve (dashed lines) overlaps nicely with the experimental points, allowing an accurate estimation of the model quantities A_∞^*, A_0 and k_R^*.

The model parameters are summarized in Table S4.

Table S4. Thermal relaxation parameters and fitting results.

Compound	Parameter	Value	St. Error	Fitting Results	Value
**AB.Me**	A_∞^*	0.93734	0.00317	**R^2**<br>**RSS**	0.9992<br>0.00076
	A_0	0.13132	0.00517		
	k_R^*	0.07002 h^{-1}	0.00112		
**AB.OMe**	A_∞^*	0.8322	0.00526	**R^2**<br>**RSS**	0.99794<br>0.00137
	A_0	0.159	0.00673		
	k_R^*	0.05901 h^{-1}	0.00165		
**AB.CO_2Me**	A_∞^*	0.88657	0.00477	**R^2**<br>**RSS**	0.99856<br>0.0011
	A_0	0.21938	0.00501		
	k_R^*	0.04294 h^{-1}	0.00099		

4.3. Fitting performance

Regarding the fitting performances, some considerations can be done.

For both investigated models, the coefficient of determination, R^2, for the three datasets is quite high, suggesting that the two models explain the mainly variability of the response data around its mean.

Finally, the residual sum of square (RSS) shows – in all the three fitting runs – a value close to zero, thus indicating that the datasets are reasonably well represented by the selected kinetic model.

5. DFT simulations

5.1 Computational details

Computational analysis has been carried out by using first principles simulations based on density functional theory (DFT), as implemented in the QuantumEspresso suite [S3]. A van der Waals corrected functional (vdw-df2-b86r [S4]) is used to optimize the geometry of the systems in the E and Z conformations, while a hybrid functional (HSE06 [S5]) is used describe the electronic structure of the resulting optimized geometries. Atomic coordinates were relaxed until all force components were smaller than 0.03 eV/Å. Ultrasoft pseudopotentials of the Vanderbilt type [S6] are used to describe the electron-ion interaction for each chemical species. Single particle wavefunctions (charges) are expanded in planewaves up to an energy cutoff of 30 Ry (300 Ry), respectively. Isolated molecules are simulated in cubic cells of 30.0 Å of size, which include a sufficient vacuum space to avoid spurious interactions among replica. The Brillouin zone of the reciprocal lattice was sampled at the Γ point.

5.2 DFT analysis

Ground state of all azobenzene-derived molecules have been evaluated through a total-energy-and-forces minimization procedure. The final total energies of *E* isomers are always lower (i.e. more stable configuration) than the Z ones. The numerical values are summarized in Table S5 (unfunctionalized pristine azobenzene was included for comparison) .

Table S5. Total energy of azobenzene derivatives in *E* and *Z* configurations.

	**Isomer *E***	**Isomer *Z***
**AB**	-188.1780003244 Ry	-188.1396345584 Ry
**AB.Me**	-261.4194234596 Ry	-261.3791013955 Ry
**AB.OMe**	-325.1457693207 Ry	-325.1055627704 Ry
**AB.CO_2.Me**	-411.7971856261 Ry	-411.7572072099 Ry

The electronic structures of the optimized molecules have been evaluated at the hybrid HSE level of theory. The results are summarized in Figure S2, which displays the total density of states (DOS), and in Table S4 which collects the energy bandgap and the energy of the HOMO and LUMO energy.

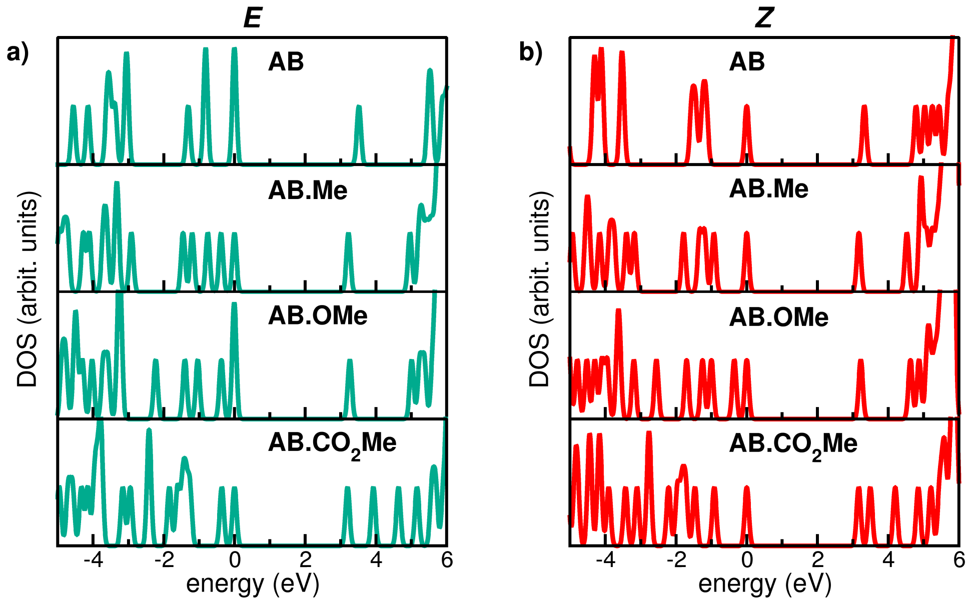

Figure S9. Density of states (DOS) of azobenzene derivatives in *E* (a) and *Z* (b) configuration. Zero energy reference is set to HOMO state of each system.

Table S6. Bandgap (Eg) and energy level of HOMO and LUMO states of azobenze derivatives in *E* (a) and *Z* (b) configuration. Zero energy reference is set to the vacuum level.

	Isomer *E*			Isomer *Z*		
	E_g	HOMO	LUMO	E_g	HOMO	LUMO
**AB**	3.52 eV	-6.33 eV	-2.81 eV	3.33 eV	-5.89 eV	-2.57 eV
**AB.Me**	3.22 eV	-5.73 eV	-2.51 eV	3.17 eV	-5.51 eV	-2.34 eV
**AB.OMe**	3.26 eV	-5.76 eV	-2.50 eV	3.22 eV	-5.53 eV	-2.30 eV
**AB.CO2.Me**	3.20 eV	-6.06 eV	-2.87 eV	3.17 eV	-5.81 eV	-2.65 eV

6. References

S1. Ślusarek J.; Nowoświat A.; Olechowska M. Logistic Model of Phase Transformation of Hardening Concrete. *Materials*, **2022**, *15*, 4403 .

S2. Jerca V.V.; Jerca F.A.; Rau I.; Manea A.M.; Vuluga D.M.; Kajzar F. Advances in understanding the photoresponsive behavior of azobenzenes substituted with strong electron withdrawing groups. *Opt. Mater.* **2015**, *48*, 160-164.

S3. Giannozzi P.; Baroni S.; Bonini N.; Calandra M.; Car R.; Cavazzoni C.; Ceresoli D.; Chiarotti G.L.; Cococcioni M.; Dabo I.; Dal Corso A.; Fabris S.; Fratesi G.; de Gironcoli S.; Gebauer R.; Gerstmann U.; Gougoussis C.; Kokalj A.; Lazzeri M.; Martin- Samos L.; Marzari N.; Mauri F.; Mazzarello R.; Paolini S.; PasquarelloA.; Paulatto L.; Sbraccia C.; Scandolo S.; Sclauzero G.; Seitsonen A.P.; Smogunov A.; Umari P.; Wentzcovitch R.M. QUANTUM ESPRESSO: a modular and open-source software project for quantum simulations of materials *J. Phys.: Condens. Matter.* **2009**, *21*, 395502.

S4.Berland K.; Cooper V.R.; Lee K.; Schröder E.; Thonhauser T.; Hyldgaard P.; Lundqvist B.I. van der Waals forces in density functional theory: a review of the vdW-DF method. *Rep. Prog. Phys.* **2015**, *78*, 066501.

S5. Heyd J.; Scuseria G.E.; Ernzerhof M. Hybrid functionals based on a screened Coulomb potential. *J. Chem. Phys.* **2003**, *118*, 8207-8215.

S6. Vanderbilt D. Soft self-consistent pseudopotentials in a generalized eigenvalue formalism. *Phys. Rev. B*, **1990**, *41*, 7892-7895.
